# The composition of the bacterial communities collected from the PM_10_ samples inside the Seoul subway and railway station

**DOI:** 10.1038/s41598-023-49848-x

**Published:** 2024-03-18

**Authors:** Shambhavi Sharma, Muhammad Jahanzaib, Ahtesham Bakht, Min-Kyung Kim, Hyunsoo Lee, Duckshin Park

**Affiliations:** 1 Department of Transportation Environmental Research, Korea Railroad Research Institute (KRRI), Uiwang, 16105 Republic of Korea; 2 Transportation System Engineering, University of Science and Technology (UST), Daejeon, 34113 Republic of Korea; 3 Kumoh National Institute of Technology (KIT), 61 Daehak-ro, Gumi-si, Gyeongsangbuk-do 39177 Republic of Korea

**Keywords:** Microbiology, Environmental sciences

## Abstract

Health implications of indoor air quality (IAQ) have drawn more attention since the COVID epidemic. There are many different kinds of studies done on how IAQ affects people’s well-being. There hasn’t been much research that looks at the microbiological composition of the aerosol in subway transit systems. In this work, for the first time, we examined the aerosol bacterial abundance, diversity, and composition in the microbiome of the Seoul subway and train stations using DNA isolated from the PM_10_ samples from each station (three subway and two KTX stations). The average PM_10_ mass concentration collected on the respective platform was 41.862 µg/m^3^, with the highest average value of 45.95 µg/m^3^ and the lowest of 39.25 µg/m^3^. The bacterial microbiomes mainly constituted bacterial species of soil and environmental origin (e.g., *Acinetobacter, Brevundimonas, Lysinibacillus, Clostridiodes*) with fewer from human sources (*Flaviflexus, Staphylococcus*). This study highlights the relationship between microbiome diversity and PM_10_ mass concentration contributed by outdoor air and commuters in South Korea’s subway and train stations. This study gives insights into the microbiome diversity, the source, and the susceptibility of public transports in disease spreading.

## Introduction

The ongoing pandemic has drawn researchers’ attention to the need to better understand microbial dynamics behavior in urban contexts. Particulate Matter (PM) is not a single type of air pollution; instead, it can operate as a vehicle to enter germs, heavy metals, and polycyclic aromatic hydrocarbons into the body. Numerous research have examined the possible effects of PM on the atmosphere and humans^1–7^. Microorganisms make up around 10% of all aerosol particles. In particular, > 80% of PM_10_ particles may be accounted by airborne bacteria with an abundance of 10^−5^ to 10^−6^ cells m^−3^ in the near-surface environment^8–10^.

Subway networks serve as a daily contact interface for many metropolitan residents. Daily commuters bring commensal bacteria and touch with organisms and mobile materials in the environment, demanding additional evaluation of the IAQ and commuter health^11–13^. The lack of air circulation, microbial contamination, and high relative humidity in the subway system cause occupational disorders such as allergic reactions and asthma attacks^14^. Several investigations on the concentration and chemical composition of subway particulate matter (PM) on station platforms, tunnels, and inside the subway have been conducted to date^15–17^. Even though aerobial microbes harms human health^18–23^, there have been few kinds of research on the biological component of PM. As a result, there is a deficit in scientific information about microbial ecology with which the worldwide human population interacts often. Human commensal microbiomes have also been found to differ depending on culture, and therefore geographically isolated studies are confined to the locations where they are conducted and miss essential differences^24^.

Except for a few kind of research that use advanced molecular approaches to define the aerobial microbiome, most investigations rely on culture-based methodology^25–28^. New York^29^, Singapore^30^, Hong Kong^22^, Barcelona^31^, Oslo^32^, and Moscow^33^ have all recently done investigations employing high throughput sequencing (HTS) in their subway systems (Table 1). Most of these initiatives have only looked at a few cities several times (Table 1). In underground environments, there is a significant gap between the highly unexplored aerial microbiota. Because hundreds of people utilize subway stations regularly, mapping the microbiome is essential^34^. Using 16S rRNA gene sequencing, we examine the current study’s air samples obtained from several Seoul subway stations (Fig. S1). This is the first experimental study to examine the diversity of the bacterial microbiome of Seoul’s train and subway stations using DNA extracted from PM_10_ air filters. This study aims to compare air bacterial microbiomes collected from PM_10_, identify uncommon and widespread species, and measure the diversity differences between two sample matrices. In conclusion, by providing the first microbial characterization of PM_10_ in Seoul’s subway and train and by highlighting the main contributing sources, including outdoor air, commuters, and soil, this study contributes to the expanding literature of research on the investigation of the microbiome in urban transportation networks. Another conclusion of this study is the susceptibility of public transportation to the spread of airborne infections. We also compared the microbial diversity collected from the subway and train stations. This research shed light on the potential association between the number of airborne bacteria and PM_10_ concentration, as well as the overall bacterial microbiomes.

**Table 1:** Analysis of few earlier Bioaerosol studies in metropolitan subway systems.

City	References	Analyzed microbiome	Method used
Seoul	Cho et al.^38^	Fungi	Culture-based
	Hwang et al.^51^	Bacteria	Culture-based
	Kim et al.^56^	Bacteria & Fungi	Culture-based
	Hwang and Park^57^	Bacteria	Culture-based
	Hwang and Cho^58^	Fungi	Culture-based
	Hwang et al.^59^	Bacteria	Culture-based
	Heo and Lee^60^	Bacteria	Culture-based
New York	Robertson et al.^29^	Bacteria, Archaea & Eukarya	DAPI counts, 454 pyrosequencing of 16S rRNA gene & Sanger
Singapore	Coleman et al.^30^	Viruses	Real-time RT-PCR/PCR
Barcelona	Triadó-Margarit et al.^31^	Bacteria, A. Fumigatus Influenza A & B and Rhinoviruses	454 Pyrosequencing of 16S rRNA gene & quantitative PCR
Moscow	Alexeev et al.^61^	Bacteria	High throughput amplicon sequence of 16S rRNA gene
Oslo	Dybwad et al.^28^	Bacteria	Culture-based, MALDI-TOF MS & 16S rRNA gene sequencing from isolates
	Dybwad et al.^26^	Bacteria	Culture-based & MALDI-TOF MS
	Gohli et al.^32^	Bacteria	Iilumina Miseq of 16S rRNA gene & quantitative PCR
Athens	Grydaki et al.^34^	Bacteria & Fungi	Internal Transcribed Spacer (ITS) region and high-throughput amplicon sequence of 16S rRNA gene

## Results

### PM_10_ concentration and bacterial 16S rRNA gene abundance

The average PM_10_ mass concentration, based on the mean values for each sampling period for all the sampled stations, was 41.862 µg/m^3^. The highest concentration was found at Yongsan station with a value of 45.95 µg/m^3^, whereas the lowest concentration, 39.25 µg/m^3^, was for the Euljiro-1-ga station (Fig. S3).

The number of bacterial 16S rRNA genes per m^3^ of air varied between 6.83 × 10^5^ and 5.31 × 10^4^. The results for PM_10_ concentration bacteria (mean = 2.58 × 10^5^ 16S rRNA genes/m^3^ of air). The abundance of the 16S rRNA gene had no significant connection with overall PM_10_ mass concentration (Pearson’s coefficient r = 0.42, *P* = 0.41 > 0.05) or any of the environmental parameters studied (Pearson’s coefficient test, *P* > 0.05). Only a few species of bacteria from the top 15 genera, including *Tissierella* and *Pseudomonas*, showed a strong correlation with PM_10_ concentrations (Fig. S7).

### Aerosol bacterial composition

**Figure 1:**
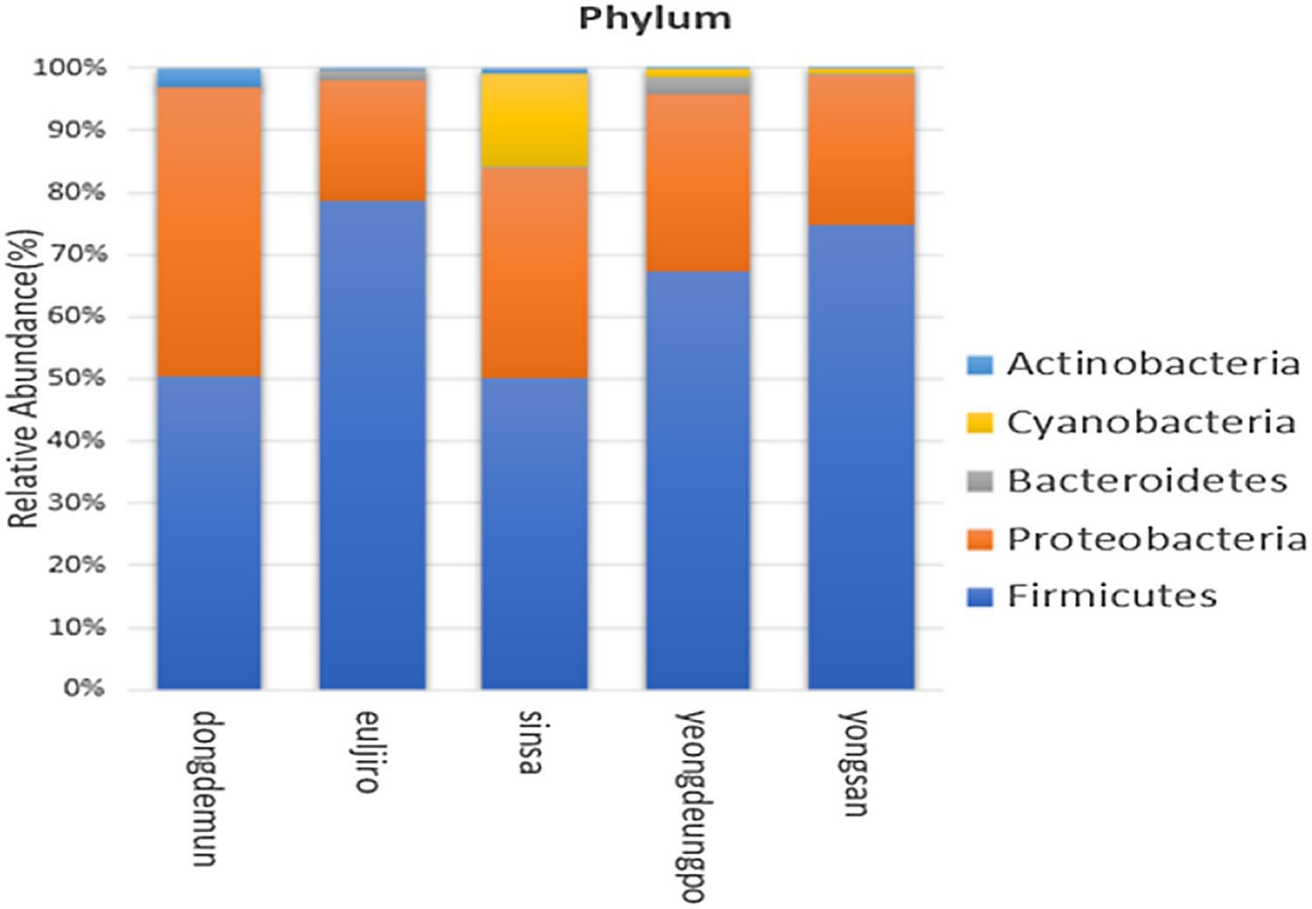
The relative abundance of bacterial OTUs at the phylum level for each PM_10_ sample taken from Seoul subway stations.

**Figure 2:**
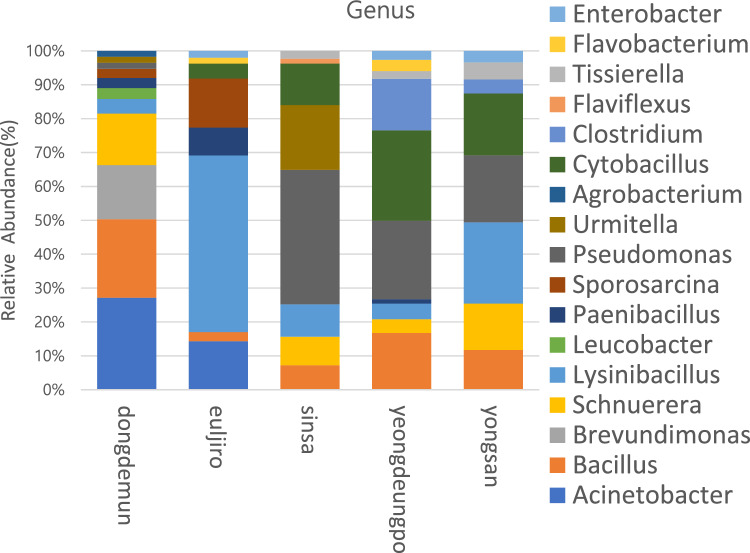
The relative abundance of bacterial OTUs at the genus level for each PM_10_ sample taken from Seoul subway stations.

*Firmicutes* (64.22%), *Proteobacteria* (30.38%), *Bacteroidetes* (1.02%), *Cyanobacteria* (3.39%), and *Actinobacteri*a (0.99%) were the top five phyla in the bacterial 16S rRNA libraries, accounting for > 90% of total reads (Fig. 1). Bacilli (45.812%), *Gammaproteobacteria* (25.238%), *Tissierellia* (13.078%), *Clostridia* (5.6975%), and *Alphaproteobacteria* (5.142%) were the most abundant classes among the 12 discovered bacterial classes with mean relative abundance > 0.1 percent across all samples (Fig. S6). There were approximately 135 genus groupings with the mean relative abundance (> 0.1 percent) across all the samples, with *Lysinibacillus* with maximum abundance. In this article, the top 15 genera are discussed (Fig. 2). *Paraclostridium, Solibacillus, Pantoea, Psychrobacillus, Peribacillus, Viridibacillus, Crocosphaera, Stenotrophomonas, Rhizobium,* and *Loltiidibacillus* were also discovered, which were not mentioned in Fig. 1 as the overall mean relative abundance of these genera were lesser than < 1%.

### Aerosol bacterial diversity

Nearly 19.87% of the total identified bacterial Operational Taxonomic Units (OTUs) were found in the overall microbiome across all five subway stations. The most common bacterial OTUs found during 16S rRNA gene sequencing are shown in Fig. 2. For bacterial species acquired, Shannon’s Diversity Index was used to determine alpha-diversity, which ranged from 3.92 (Euljiro station sample) to 6.33 (Yeongdungpo station sample) and from 4.32 (Euljiro station sample) to 5.97 (Yongsan station sample) for PM_10_ bacterial samples (Fig. S4).

**Figure 3:**
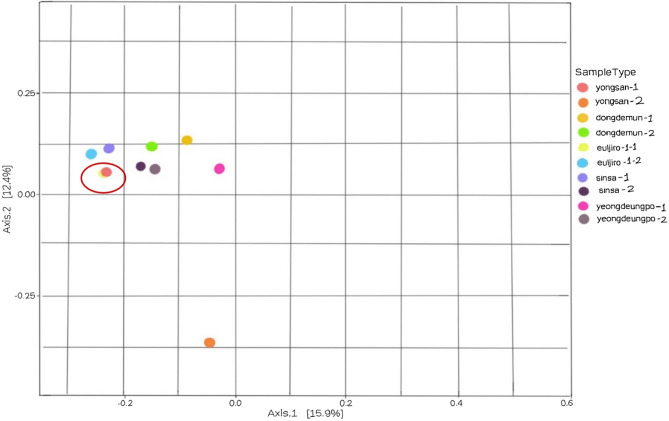
The Bray–Curtis dissimilarity and Jaccard Distance are used to visualize beta diversity in principle coordinate analysis (PCoA).

The clustering (and potential separation) of bioaerosol samples taken from filters was assessed using principal coordinate analysis (PCoA). The samples obtained from the filter of Euljiro-1-ga and Yongsan-1 were the most similar. In contrast, all the other samples were closer, except for the sample acquired from Yongsan-2, which was the most dissimilar compared to the different samples (Fig. 3).

### The most abundant bacterial OTUs’ associated environments

Table 2 lists the 15 topmost abundant bacterial OTUs (averaged samples from all stations) with their corresponding habitats, which indicate likely source settings. According to the data for top bacterial genera, the most prevalent OTUs indicated different source environments, with the bulk coming from soil sources. Water, sediments, riverbeds, air dust, debris, and human sources are among the other causes (Table 2).

**Table 2:** The top 15 most enriched bacterial genera and their likely sources are shown below.

Bacterial genus	Possible source
*Brevundimonas*	Soil, aquatic habitat, deep subsea sediment
*Bacillus*	Soil, air dust, debris
*Lysinibacillus*	Soil
*Enterobacter*	Soil, water, sewage
*Pseudomonas*	Soil, water, plants
*Tissierella*	Soil, humans
*Cytobacillus*	Soil, marine segments, living organisms
*Sporosarcina*	Soil, animal sources
*Paenibacillus*	Soil, water, sewage, sediments
*Clostridioides*	Soil, water, animals
*Flavobacterium*	Soil, animals, river sediments
*Flaviflexus*	humans
*Schnuerera*	Water, soil, sediments
*Urmitella*	Soil, humans
*Acinetobacter*	Soil, wetland, riverbed

### Correlation between bacterial diversity and PM_10_ concentration

**Figure 4:**
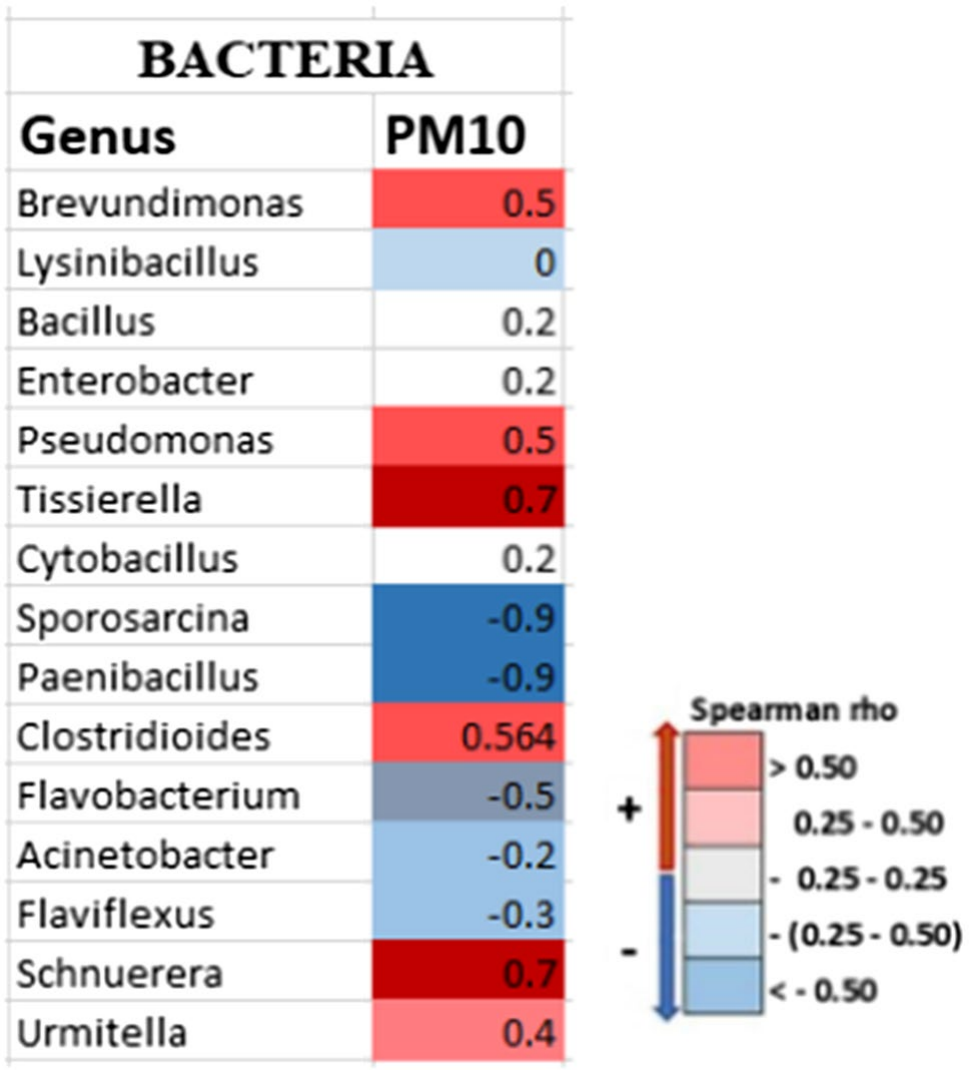
The relationship between the relative gene abundance of the Top 15 most abundant genera collected from filter and PM mass concentration was calculated using Spearman’s rank correlation coefficient rho (ρ).

For possible correlations, the top 15 most abundant bacterial taxa recovered from the filter were compared to the average values of the concurrently recorded PM_10_ mass level (Fig. 4). Several genera, including *Brevundimonas, Pseudomonas, Tissierella, Clostridioides,* and *Schnuerera,* showed a positive and strong positive association (> 0.5) with PM_10_ concentration. In contrast, other genera, such as *Sporosarcina, Paenibacillus, Flavobacterium, Flaviflexus,* and *Actinobacteria,* showed a negative correlation (Fig. 4).

## Discussion

The significance of public transportation networks cannot be understated, as they are used by the general population daily and can be a source of infectious disease and bioterrorism^35–37^. Even though particulate matter includes micro-biological parts, most bioaerosol research focuses on the total suspended particulate matter^29,31^ Nevertheless, microbial features are rarely included in bioaerosol studies. Microbial components are, however, infrequently considered in bio-aerosol studies. This is because biological and biological components of airborne particles originate from different locations, which could lead to misunderstandings about PM level. The disparity in origin of locations between biological and biological components of airborne particles contributes to variations in PM levels. Using 16S rRNA gene sequencing, this is the first pilot study of the bacterial microbiome in the Seoul subway.

Numerous culture-based methods have been used to investigate the Seoul subway’s microbiome (Table 1). Several studies around the world have used 16S rRNA sequencing in recent years (Table 1), but there is relatively little information about overall subway bioaerosol levels. Our research looked at the bacterial microbiome in various sites and collected PM from filters. We also discovered that there was no statistical difference when various bacterial populations were sampled from different sites inside the same station. We compared the biological composition of the PM_10_ as well as the bacterial communities directly.

Compared to the subway station platforms, the KTX station platforms had a greater concentration of average PM_10_ than the station itself. This may be because the KTX stations have more passenger inflow and outflow as they include both KTX and subway stations. Our findings are similar to those reported from other subway systems worldwide. Based on qPCR for samples taken inside trains, on platforms, and in lobbies^34^, the average bacterial load reported for the Barcelona subway system (4.46 × 10^4^ equivalent E. coli genomes/m^3^ of air) was comparable to the mean bacterial concentration found in the Seoul Metro station (3.78 × 10^5^ 16S rRNA genes/m^3^ of air equivalent to 4.03 × 10^4^ E. coli genome. Based on fluorescence microscopy, ^34^ calculated an average value of 2.2 × 10^4^ cells/m^3^ for the overall airborne microbial load of multiple New York subway platforms. However, comparing several research outcomes requires caution since quantitative measurements are incomparable when sample collection and processing characteristics, such as assay design and efficiency, vary. Furthermore, unlike other bioaerosol molecular investigations^29,31^, where total suspended particulate matter has traditionally been analyzed, the bacterial abundance calculated from the filter here represents the inhalable component PM_10_ rather than it.

The major bacterial groups were also from Firmicutes, Proteobacteria, Bacteroides, and Actinobacteria in studies conducted using HTS in Athens Subway^34^, Oslo subways^32^, Barcelona^31^, Hong Kong^22^, and New York^29^. At the genus level, *Enterobacter, Flavobacterium, Tissierella, Flaviflexus, Clostridium, Cytobacillus, Agrobacterium, Urmitella, Pseudomonas, Sporosarcina, Brevundimonas, Bacillus, Acinetobacter, Schnuerera, Lysinibacillus,* and *Leucobacter* dominated the Seoul bacterial microbiome, accounting for 28.5 percent of the total sources. Similar to studies conducted in the Athens metro^34^ and Moscow Subway^33^, the bacterial microbiome is dominated by non-human associated taxa, primarily soil enriched bacteria, followed by water and sediments in outdoor sources. In contrast, the New York subway platforms were found to be dominated by bacterial genera associated with both terrestrial/ aquatic environments (e.g., *Acinetobacter, Arthrobacter*)^29^.

Similarly, the most prevalent bacterial species discovered while commuting in Hong Kong’s subway system were those connected with human skin (*Corynebacterium, Micrococcus, Propionibacterium, Enhydrobacter, Staphylococcus*). On the other hand, environmental bacteria (*Sphingobium, Xanthomonas,* and *Blastomonas*) were among the most regularly found airborne microbes^22^. The present study discovered major genera previously identified in the air of Oslo subway stations (e.g., *Staphylococcus, Micrococcus, Sphingomonas,* and *Hymenobacter*)^31,32^. discovered that the proportions of human-related bacteria (*Corynebacterium, Staphylococcus, Neisseria,* and *Enhydrobacter*) in the Barcelona subway (various settings) were below 1% and that aerosols in the Barcelona subway (multiple settings) were more highly enriched with environmental taxa (e.g., *Methylobacterium, Paracoccus, Bradyrhizobium,* and *Chitinophagaceae*). Our results showed that the subway’s air microbiomes were diverse in terms of bacteria. However, the cumulative relative abundance of bacterial genera (> 1%) connected with species revealed in our results demonstrates that airborne bacterial communities, which were dominated by a few widespread taxa, were more related to the presence of abiotic factors such as soil and water with *Lactobacillus, Enterobacter,* and *Enterococcus*, as well as specific communities from the human origin^25,34, 38–41^.

Human habitation is one of the most prevalent sources of airborne microbial particles in the interior environment^42–44^. According to various studies, outdoor air appears to be a pivotal contribution to the airborne microbial diversity in the Metro station^31,45^. Ventilation strategies have been proven to alter the microbial makeup of the built environment^41,46^. According to^47^, introducing unfiltered air into a well-ventilated occupied area has a considerable impact on indoor bioaerosol microbial diversity. It tends to increase the composition similarity between indoor and outdoor air microbiota. Additionally, prior bioaerosol studies in subways based on sequencing amply demonstrate that outside air is the primary driver of subway aerosol microbial composition^22,29^.

Furthermore, the visible airborne microbiome is more diverse in naturally ventilated interior spaces than in mechanical ventilation conditions^41^. Because soil particles might act as microbial transporters, the discovery of crustal matter, common in soil and road dust found outside, is compatible with the detection of soil-related bacteria (e.g., *Paracoccus, Rubellimicrobium, Sphingomonas, Arthrobacter,* and others) in the station’s air. While there is some air mixing between the outside and the subway station, we hypothesize that other influences also shaped the microbial population in the underground. In the Seoul subway, in particular, mechanical ventilation may be crucial in influencing microbial ecosystems. The sort of ventilation system used within the Seoul subway is of the Programmable Logic Controllers (PLC) variety.

It is a programmed-based ventilation system dependent on the number of cabins. Therefore, it is only operational during rush hours and not all day. The two KTX stations are operated primarily outside and have natural ventilation predominating on the station platforms. These aboveground lines displayed community distinctions in contrast to the mechanically ventilated, underground interior stations. Screen doors along the platforms of the indoor and subterranean stations would prevent adequate airflow between the passenger platform areas and the tunnel track on these lines^22,48^. The tunnel region would be mostly filled with external air from the intake ventilation systems, but due to the screen doors, the platform area would only get minor outdoor air while a train is stopped. Because most aboveground platforms are open to ambient air, above-ground lines are less susceptible to this limited impact of external air.

The use of screen doors may further limit the amount of external air that can reach the subterranean platform areas because our integrated sampling method includes air samples obtained from the platforms. Additionally, although more details, including station depths, were not gathered, it has been demonstrated that communities at deeper underground stations are more diverse^48–51^. Therefore, more research on the connections between these variables and microbial assemblage changes in interior settings can help us understand the dynamics of the microbial community in this particular indoor environment.

## Materials and methods

### Sampling site and process

This study was conducted in December 2021 since winters in Korea have exceptionally high outdoor PM concentrations. Five stations were chosen, including two Korea Train Express (KTX) stations (Yeongdeungpo and Yongsan), as well as Dongdaemun, Euljiro-1-ga and Sinsa Stations, as they are all part of the Seoul Subway station (Fig. S1). In this study, subway stations are underground and have at least one exit. The subway stations have separate facing platform with fully sealed screen door and without any air purifiers. The ventilation system is PLC controlled mechanical ventilator which can be controlled via programming. The selected subway stations are hubs for the most significant passenger movement and transfer. The KTX stations were chosen along with subway stations because they share a platform with a train station and a subway station, resulting in a more significant number of passengers and more diversified microbial populations. A mini volume air sampler (Model PAS201, Air Metrics Co.) was used to gather PM_10_ samples during the investigation. Before and after sampling, a membrane filter (PTFE Membrane Filter 0.2 µm 47 mm, Whatman) was used to filter the flow at a rate of 5 L/min. The samples were measured using an electronic scale (Model HM-202, A&D Co.) with a 0.01 mg sensitivity after being kept in an electronic desiccator (Model Oyin 09678BN, Sanplatec Co.) for three days at a constant temperature and humidity level. To account for impurities encountered during sample collection and processing, one unexposed filter (field blank) was also examined. The AirScan equipment (Fig. S2) from Sensoronic was also additionally used to measure real-time PM_10_ mass concentrations (Fig. S3).

Two samples from each station, were collected making it for 10 samples. One sample for rush hour and one sample for normal hours were taken. A blank filter was also put at each station to assess pollutants exposed during the sample collection and processing.

The filter samples were carefully wrapped in bags and delivered to the laboratory for both the gravimetric and microbial analysis. As mentioned by^30^, air samples were taken, and DNA from air filters was extracted. First, 70% ethanol was used to sterilize all of the equipment. Each polytetrafluoroethylene (PTFE) filter was taken out of the sampler, put in a 50 mL Falcon tube, and vortexed for 5 s while still dry. Each 50 mL Falcon tube holding a filter was then filled with one mL of a 0.5% BSA solution, and the tubes were vortexed once more for 15 s. Each 1.5 mL conical tube from the samplers received one mL of a 0.5% Bovine Serum Albumin (BSA) solution and was vortexed for 10 s. The BSA solutions from the 50 mL Falcon tubes including filters were combined with the corresponding 1.5 mL conical tube sample using cryotube vials. Each 15 mL Falcon tube from the samplers was filled with 2 mL of BSA solution, vortexed for 15 s, and then put into cryotube vials.

The additional methodology details the procedure (Fig. S2). After this, DNA extraction was carried out using the MagMAX cell-free total nucleic acid kit (purchased from ThermoFisher Scientific), carefully following the kit’s instructions. The isolated DNA was kept at − 20 °C until 16S rRNA sequencing.

### DNA isolation and sequencing of 16S rRNA

To calculate the bacterial abundance, 16S rRNA gene sequencing was performed. Using the 16S metagenomic sequencing library preparation methods, a 16S rRNA sequencing library targeting the V3 and V4 hypervariable regions of the 16S rRNA gene was produced (Illumina, San Diego, CA, USA). KAPA HiFi HotStart ReadyMix (KAPA Biosystems, Wilmington, MA, USA) and Agencourt AMPure XP system (Beckman Coulter Genomics, Brea, CA, USA) were used for PCR and purification of the PCR product, respectively. The initial PCR used a 12 ng template and region-specific primers that were found to work with Illumina index and sequencing adapters. (forward primer: 5′ TCGTCGGCAGCGTCAGATGTGTATAAGAGACAGTCGTCGGCAGCGTCAGATGTGTAT AAGAGACAGCCTACGGGNGGCWGCAG-3′; reverse primer: 5′ GTCTCGTGGGCTCGGAGATGTGTATAAGAGACAGGTCTCGTGGGCTCGGAGATGTGT ATAAGAGACAGGACTACHVGGGTATCTAATCC-3′). After magnetic bead-based purification of PCR products, the second PCR was performed with primers from an Illumina Nextera XT Index Kit with a restricted cycle. Then, purified PCR products were seen using gel electrophoresis and quantified using a Qubit 3.0 fluorometer and a Qubit dsDNA HS Assay Kit (Thermo Scientific). An Agilent 2100 bioanalyzer was used to check the quality of the pooled samples before sequencing (Agilent). Libraries were measured using the CFX96 Real-Time System and qPCR (Biorad). After normalization, the resulting library was sequenced on the Illumina Miseq system using 300 bp paired-end reads.

### Statistical analysis

The AirScan equipment (Fig. S2) from Sensoronic was also used to measure real-time PM_10_ mass concentrations (Fig. S3). R programming language, version 3.6.0^52^, and MATLAB 2021a were used to analyze the data. Each filter determined the time-integrated estimates of 16S rRNA gene abundance by qPCR. The mean values of PM_10_ data acquired from each station were also compared. Non-parametric tests were used to examine the normality of data distribution when the conditions for parametric counterparts were not met. A t-test was used to compare group means of PM_10_ mass and qPCR quantification results from the filter (Statistical significance for the test was set at *P* < 0.05). The Pearson correlation coefficient was used to determine the relationship between bacterial abundance, PM_10_ mass concentration, and environmental parameters (temperature and relative humidity). Spearman’s rank correlation was used to establish a link between PM_10_ mass concentration and relative gene abundance of the dominating bacterial taxa.

The STAMP software suite^53^ was used to evaluate the taxonomic profiles of bacterial samples. The taxonomic composition of the bacterial species was determined using the R programming language, version 3.6.0, and the Naive Bayes Classifier implemented in QIIME-2 (^54^; R Core Team 2013). The Chao1, Shannon diversity matrix was calculated using QIIME-2 to assess alpha diversity. The incidence-based Jaccard index and Bray–Curtis dissimilarity were used to measure beta diversity (dissimilarity in microbial composition among samples) using Phyloseq. Principal Coordinates Analyses (PCoA) 2D plots made with the ggplot2 software^39^ within phyloseq were used to depict the collected distance matrices. Using permutation-based multivariate analysis of variance (PERMANOVA)^55^ with 9999 permutations, the comparison categories were used to find statistical differences in bacterial composition among filter samples. Within QIIME, statistical significance was deter-mined using *P* < 0.05.

### Ethical approval

All authors give consent for the publication of the manuscript and the materials incorporated.

## Conclusions

Given their role in facilitating interactions between human and other bacterial microbiomes, as well as their role in infectious disease transmission and bioterrorism risk, understanding the composition and dynamics of air microbiomes in mass transit environments is essential for future developments in human health and security. Using samples from filters, our pilot study analyzes the biological composition of PM_10_ in the Seoul Subway. Our findings pave the path for far more comprehensive subway air quality studies beyond broad physicochemical aerosol characterization.

The bacterial diversity gathered using the filter, matches our findings. Overall, we discovered that environmental bacteria dominate the aerial bacterial microbiome, with soil serving as the primary source and human commensal bacteria contributing less. Our findings also reveal information about numerous bacterial communities linked to PM_10_ mass concentration. Finally, our results provide important information about the microbial communities associated with public transportation, which can be used to understand better the elements that affect biosafety and critical infrastructure security.

### Supplementary Information

Supplementary Information 1.

Supplementary Information 2.

## Data Availability

The datasets generated during the study will be available from the corresponding author on reasonable request. The datasets generated and/or analysed during the current study are available in the supplementary material part of this manuscript.
