# Supplementary material for: The composition of the bacterial communities collected from the PM10 samples inside the Seoul subway and railway station
Source: Sci Rep. 2024 Mar 18;14:6478. doi: 10.1038/s41598-023-49848-x (PMC10948816; doi:10.1038/s41598-023-49848-x)
Supplement: Supplementary file 1 — Supplementary Information 1. [file 41598_2023_49848_MOESM1_ESM.pdf]

| Taxonomy | Kingdom | Phylum | Class | Order | Family | Genus | Species | Strain |
|---|---|---|---|---|---|---|---|---|
| k__Bacteri | Bacteria | Firmicutes | Bacilli | Bacillales | Bacillaceae | Bacillus | cereus | Bacillus cer |
| k__Bacteri | Bacteria | Firmicutes | Bacilli | Bacillales | Bacillaceae | Lysinibacill | sphaericus | Lysinibacill |
| k__Bacteri | Bacteria | Firmicutes | Bacilli | Bacillales | Bacillaceae | Lysinibacill | telephonicu | Lysinibacill |
| k__Bacteri | Bacteria | Firmicutes | Bacilli | Bacillales | Bacillaceae | Bacillus | pumilus | Bacillus pu |
| k__Bacteri | Bacteria | Proteobact | Gammapro | Moraxellal | Moraxellac | Acinetobac | pittii | Acinetobac |
| k__Bacteri | Bacteria | Proteobact | Gammapro | Pseudomo | Pseudomo | Pseudomo | stutzeri | Pseudomo |
| k__Bacteri | Bacteria | Proteobact | Gammapro | Moraxellal | Moraxellac | Acinetobac | lwoffii | Acinetobac |
| k__Bacteri | Bacteria | Firmicutes | Bacilli | Bacillales | Bacillaceae | Bacillus | anthracis | Bacillus ant |
| k__Bacteri | Bacteria | Firmicutes | Tissierellia | Tissierellal | Tissierellac | Schnuerera | ultunensis | [Clostridiu |
| k__Bacteri | Bacteria | Firmicutes | Bacilli | Bacillales | Bacillaceae | Cytobacillu | depressus | Bacillus de |
| k__Bacteri | Bacteria | Firmicutes | Bacilli | Bacillales | Bacillaceae | Bacillus | aerius | Bacillus ae |
| k__Bacteri | Bacteria | Firmicutes | Bacilli | Bacillales | Paenibacill | Paenibacill | illinoisensis | Paenibacill |
| k__Bacteri | Bacteria | Proteobact | Gammapro | Pseudomo | Pseudomo | Pseudomo | cedrina | Pseudomo |
| k__Bacteri | Bacteria | Proteobact | Gammapro | Moraxellal | Moraxellac | Acinetobac | radioresist | Acinetobac |
| k__Bacteri | Bacteria | Firmicutes | Clostridia | Eubacterial | Clostridiac | Clostridium | subtermina | Clostridium |
| k__Bacteri | Bacteria | Firmicutes | Tissierellia | Tissierellal | Tissierellac | Tissierella | praeacuta | Tissierella p |
| k__Bacteri | Bacteria | Firmicutes | Bacilli | Bacillales | Bacillaceae | Bacillus | chungange | Bacillus chu |
| k__Bacteri | Bacteria | Proteobact | Gammapro | Moraxellal | Moraxellac | Acinetobac | populi | Acinetobac |
| k__Bacteri | Bacteria | Proteobact | Betaproteo | Burkholder | Comamona | Xenophilus | aerolatus | Xenophilus |
| k__Bacteri | Bacteria | Proteobact | Gammapro | Pseudomo | Pseudomo | Pseudomo | silesiensis | Pseudomo |
| k__Bacteri | Bacteria | Proteobact | Gammapro | Xanthomo | Xanthomo | Stenotroph | rhizophila | Stenotroph |
| k__Bacteri | Bacteria | Firmicutes | Bacilli | Bacillales | Bacillaceae | Bacillus | licheniform | Bacillus lich |
| k__Bacteri | Bacteria | Proteobact | Gammapro | Pseudomo | Pseudomo | Pseudomo | oryzihabita | Pseudomo |
| k__Bacteri | Bacteria | Proteobact | Alphaprote | Caulobacte | Caulobacte | Brevundim | olei | Brevundim |
| k__Bacteri | Bacteria | Firmicutes | Bacilli | Bacillales | Bacillaceae | Psychrobac | psychrodur | Psychrobac |
| k__Bacteri | Bacteria | Proteobact | Gammapro | Pseudomo | Pseudomo | Pseudomo | putida | Pseudomo |
| k__Bacteri | Bacteria | Proteobact | Betaproteo | Burkholder | Oxalobacte | Massilia | aurea | Massilia au |
| k__Bacteri | Bacteria | Firmicutes | Tissierellia | Tissierellal | Tissierellac | Urmitella | timonensis | Urmitella ti |
| k__Bacteri | Bacteria | Firmicutes | Bacilli | Bacillales | Bacillaceae | Alkalihalob | plakortidis | Bacillus pla |
| k__Bacteri | Bacteria | Proteobact | Gammapro | Enterobact | Erwiniacea | Erwinia | aphidicola | Erwinia aph |
| k__Bacteri | Bacteria | Firmicutes | Bacilli | Bacillales | Bacillaceae | Alkalihalob | hunanensis | Bacillus hu |
| k__Bacteri | Bacteria | Proteobact | Gammapro | Pseudomo | Pseudomo | Pseudomo | flavescens | Pseudomo |
| k__Bacteri | Bacteria | Proteobact | Alphaprote | Sphingomo | Sphingomo | Sphingomo | jeddahensi | Sphingomo |
| k__Bacteri | Bacteria | Firmicutes | Bacilli | Bacillales | Bacillaceae | Lysinibacill | boronitoler | Lysinibacill |
| k__Bacteri | Bacteria | Proteobact | Gammapro | Moraxellal | Moraxellac | Acinetobac | johnsonii | Acinetobac |
| k__Bacteri | Bacteria | Firmicutes | Clostridia | Eubacterial | Clostridiac | Clostridium | argentinen | Clostridium |
| k__Bacteri | Bacteria | Proteobact | Gammapro | Enterobact | Enterobact | Klebsiella | pneumonia | Klebsiella p |
| k__Bacteri | Bacteria | Firmicutes | Bacilli | Bacillales | Paenibacill | Paenibacill | lautus | Paenibacill |
| k__Bacteri | Bacteria | Proteobact | Gammapro | Pseudomo | Pseudomo | Pseudomo | luteola | Pseudomo |
| k__Bacteri | Bacteria | Firmicutes | Clostridia | Eubacterial | Peptostrep | Clostridioid | mangenoti | Clostridioid |
| k__Bacteri | Bacteria | Firmicutes | Bacilli | Bacillales | Bacillaceae | Cytobacillu | kochii | Bacillus koc |
| k__Bacteri | Bacteria | Proteobact | Gammapro | Enterobact | Enterobact | Enterobact | hormaeche | Enterobact |
| k__Bacteri | Bacteria | Actinobact | Actinomyc | Corynebact | Nocardiace | Rhodococc | qingshengi | Rhodococc |
| k__Bacteri | Bacteria | Firmicutes | Bacilli | Bacillales | Paenibacill | Paenibacill | popilliae | Paenibacill |
| k__Bacteri | Bacteria | Firmicutes | Bacilli | Bacillales | Planococca | Sporosarci | newyorken | Sporosarci |
| k__Bacteri | Bacteria | Cyanobacte | unclassified | Nostocales | Calotrichac | Dulcicaloth | desertica | Calothrix d |
| k__Bacteri | Bacteria | Firmicutes | Bacilli | Bacillales | Planococca | Sporosarci | luteola | Sporosarci |
| k__Bacteri | Bacteria | Firmicutes | Clostridia | Eubacterial | Clostridiac | Alkaliphilus | oremlandii | Alkaliphilus |
| k__Bacteri | Bacteria | Proteobact | Betaproteo | Burkholder | Comamona | Xenophilus | arsenicires | Xenophilus |

| | | | | | | | | |
|---|---|---|---|---|---|---|---|---|
| k__Bacteri | Bacteria | Proteobact | Gammapro | Moraxellal | Moraxellac | Psychrobac | pulmonis | Psychrobac |
| k__Bacteri | Bacteria | Proteobact | Gammapro | Pseudomor | Pseudomor | Pseudomor | turukhansk | Pseudomor |
| k__Bacteri | Bacteria | Proteobact | Gammapro | Xanthomor | Xanthomor | Stenotroph | bentonitica | Stenotroph |
| k__Bacteri | Bacteria | Proteobact | Betaproted | Burkholder | Alcaligenac | Achromoba | spanius | Achromoba |
| k__Bacteri | Bacteria | Firmicutes | Bacilli | Bacillales | Paenibacill | Paenibacill | glucanolyti | Paenibacill |
| k__Bacteri | Bacteria | Proteobact | Alphaprote | Caulobacte | Caulobacte | Brevundim | poindexter | Brevundim |
| k__Bacteri | Bacteria | Proteobact | Gammapro | Pseudomor | Pseudomor | Pseudomor | fulva | Pseudomor |
| k__Bacteri | Bacteria | Proteobact | Betaproted | Burkholder | Alcaligenac | Achromoba | animicus | Achromoba |
| k__Bacteri | Bacteria | Actinobact | Actinomyc | Micrococca | Sanguibact | Sanguibact | keddieii | Sanguibact |
| k__Bacteri | Bacteria | Firmicutes | Bacilli | Bacillales | Bacillaceae | Peribacillus | loiseleuriae | Bacillus lois |
| k__Bacteri | Bacteria | Firmicutes | Bacilli | Bacillales | Planococca | Viridibacill | arvi | Viridibacill |
| k__Bacteri | Bacteria | Proteobact | Gammapro | Moraxellal | Moraxellac | Acinetobac | seohaensis | Acinetobac |
| k__Bacteri | Bacteria | Proteobact | Alphaprote | Sphingomo | Sphingomo | Sphingomo | olei | Sphingomo |
| k__Bacteri | Bacteria | Proteobact | Gammapro | Enterobact | Enterobact | Enterobact | mori | Enterobact |
| k__Bacteri | Bacteria | Cyanobact | unclassified | Pleurocaps | Xenococca | Foliisarcina | bertiogensi | Foliisarcina |
| k__Bacteri | Bacteria | Proteobact | Gammapro | Xanthomor | Xanthomor | Stenotroph | chelatiphag | Stenotroph |
| k__Bacteri | Bacteria | Actinobact | Actinomyc | Micrococca | Microbacte | Microbacte | saperdae | Microbacte |
| k__Bacteri | Bacteria | Firmicutes | Tissierellia | Tissierellal | Tissierellac | Sporanaer | acetigenes | Sporanaer |
| k__Bacteri | Bacteria | Proteobact | Gammapro | Enterobact | Enterobact | Lelliottia | nimipressu | Lelliottia ni |
| k__Bacteri | Bacteria | Proteobact | Betaproted | Burkholder | Oxalobacte | Duganella | zoogloeoid | Duganella |
| k__Bacteri | Bacteria | Actinobact | Actinomyc | Micrococca | Micrococca | Rothia | koreensis | Kocuria kor |
| k__Bacteri | Bacteria | Proteobact | Gammapro | Moraxellal | Moraxellac | Moraxella | osloensis | Moraxella |
| k__Bacteri | Bacteria | Firmicutes | Bacilli | Bacillales | Planococca | Solibacillus | kalamii | Solibacillus |
| k__Bacteri | Bacteria | Firmicutes | Clostridia | Eubacterial | Clostridiace | Paraclostri | bifermenta | Paraclostri |
| k__Bacteri | Bacteria | Bacteroide | Flavobacte | Flavobacte | Flavobacte | Flavobacte | acidificum | Flavobacte |
| k__Bacteri | Bacteria | Proteobact | Gammapro | Moraxellal | Moraxellac | Acinetobac | baumannii | Acinetobac |
| k__Bacteri | Bacteria | Firmicutes | Bacilli | Bacillales | Staphyloco | Mammaliic | sciuri | Staphyloco |
| k__Bacteri | Bacteria | Actinobact | Actinomyc | Micrococca | Micrococca | Renibacter | salmoninar | Renibacter |
| k__Bacteri | Bacteria | Firmicutes | Bacilli | Bacillales | Bacillaceae | Cytobacillu | horneckiae | Bacillus ho |
| k__Bacteri | Bacteria | Proteobact | Gammapro | Pseudomor | Pseudomor | Pseudomor | hutmensis | Pseudomor |
| k__Bacteri | Bacteria | Firmicutes | Bacilli | Bacillales | Bacillaceae | Bacillus | yapensis | Bacillus ya |
| k__Bacteri | Bacteria | Proteobact | Alphaprote | Pelagibacte | Pelagibacte | Candidatus | Pelagibacte | Candidatus |
| k__Bacteri | Bacteria | Firmicutes | Bacilli | Bacillales | Bacillaceae | Virgibacillu | pantothent | Virgibacillu |
| k__Bacteri | Bacteria | Proteobact | Gammapro | Xanthomor | Xanthomor | Pseudoxan | helianthi | Pseudoxan |
| k__Bacteri | Bacteria | Actinobact | Actinomyc | Micrococca | Micrococca | Pseudarthr | defluvii | Pseudarthr |
| k__Bacteri | Bacteria | Proteobact | Alphaprote | Hyphomicr | Rhizobiace | Rhizobium | nepotum | Rhizobium |
| k__Bacteri | Bacteria | Firmicutes | Clostridia | Eubacterial | Clostridiace | Paraclostri | benzoelytic | Paraclostri |
| k__Bacteri | Bacteria | Proteobact | Gammapro | Pseudomor | Pseudomor | Pseudomor | fluorescens | Pseudomor |
| k__Bacteri | Bacteria | Proteobact | Alphaprote | Sphingomo | Sphingomo | Sphingomo | canadensis | Sphingomo |
| k__Bacteri | Bacteria | Proteobact | Gammapro | Enterobact | Erwiniacea | Pantoea | septica | Pantoea se |
| k__Bacteri | Bacteria | Firmicutes | Bacilli | Lactobacill | Enterococc | Enterococc | hirae | Enterococc |
| k__Bacteri | Bacteria | Firmicutes | Bacilli | Bacillales | Bacillaceae | Niallia | circulans | Neobacillus |
| k__Bacteri | Bacteria | Proteobact | Gammapro | Xanthomor | Xanthomor | Stenotroph | tumulicola | Stenotroph |
| k__Bacteri | Bacteria | Actinobact | Actinomyc | Micrococca | Microbacte | Leucobacte | chromiired | Leucobacte |
| k__Bacteri | Bacteria | Firmicutes | Bacilli | Bacillales | unclassified | Exiguobact | sibiricum | Exiguobact |
| k__Bacteri | Bacteria | Proteobact | Gammapro | Enterobact | Erwiniacea | Erwinia | mallotivora | Erwinia ma |
| k__Bacteri | Bacteria | Firmicutes | Tissierellia | Tissierellal | Peptoniphi | Anaerococ | octavius | Anaerococ |
| k__Bacteri | Bacteria | Firmicutes | Tissierellia | Tissierellal | Tissierellac | Tissierella | carlieri | Tissierella |
| k__Bacteri | Bacteria | Proteobact | Gammapro | Xanthomor | Xanthomor | Stenotroph | maltophilia | Stenotroph |
| k__Bacteri | Bacteria | Proteobact | Alphaprote | Caulobacte | Caulobacte | Brevundim | diminuta | Brevundim |

| | | | | | | | | |
|---|---|---|---|---|---|---|---|---|
| k__Bacteri | Bacteria | Proteobact | Alphaprote | Hyphomicr | Parvibacula | Rhodoligot | jinshengii | Rhodoligot |
| k__Bacteri | Bacteria | Firmicutes | Bacilli | Bacillales | Bacillaceae | Virgibacillu | halodenitri | Virgibacillu |
| k__Bacteri | Bacteria | Proteobact | Gammapro | Pseudomo | Pseudomo | Pseudomo | frederiksbe | Pseudomo |
| k__Bacteri | Bacteria | Proteobact | Gammapro | Moraxellal | Moraxellac | Psychroba | submarinus | Psychroba |
| k__Bacteri | Bacteria | Firmicutes | Negativicut | Selenomon | Sporomusa | Sporomusa | sphaeroide | Sporomusa |
| k__Bacteri | Bacteria | Firmicutes | Bacilli | Bacillales | Paenibacill | Brevibacillu | halotolera | Brevibacillu |
| k__Bacteri | Bacteria | Proteobact | Gammapro | Enterobact | Enterobact | Enterobact | cloacae | Enterobact |
| k__Bacteri | Bacteria | Firmicutes | Bacilli | Bacillales | Bacillaceae | Oceanobac | kimchii | Oceanobac |
| k__Bacteri | Bacteria | Actinobact | Actinomyc | Micrococca | Microbacte | Agromyces | bauzanensi | Agromyces |
| k__Bacteri | Bacteria | Firmicutes | Bacilli | Bacillales | Bacillaceae | Neobacillus | vireti | Bacillus vir |
| k__Bacteri | Bacteria | Actinobact | Actinomyc | Micrococca | Micrococca | Arthrobact | luteolus | Arthrobact |
| k__Bacteri | Bacteria | Proteobact | Gammapro | Pseudomo | Pseudomo | Pseudomo | chlororaph | Pseudomo |
| k__Bacteri | Bacteria | Proteobact | Betaprote | Burkholder | Oxalobacte | Duganella | levis | Duganella l |
| k__Bacteri | Bacteria | Proteobact | Alphaprote | Hyphomicr | Rhizobiace | Agrobacter | fabrum | Agrobacter |
| k__Bacteri | Bacteria | Cyanobact | unclassifie | Chroococca | Aphanothe | Crocospha | watsonii | Crocospha |
| k__Bacteri | Bacteria | Firmicutes | Bacilli | Bacillales | Bacillaceae | Lederbergi | graminis | Bacillus gra |
| k__Bacteri | Bacteria | Actinobact | Actinomyc | Actinomyc | Actinomyc | Flaviflexus | salsibiostra | Flaviflexus |
| k__Bacteri | Bacteria | Proteobact | Alphaprote | Rhodobact | Rhodobact | Pararhodo | oceanensis | Pararhodo |
| k__Bacteri | Bacteria | Firmicutes | Tissierellia | Tissierella | Tissierellac | Tissierella | creatinoph | Tissierella |
| k__Bacteri | Bacteria | Actinobact | Actinomyc | Micrococca | Micrococca | Kocuria | palustris | Kocuria pal |
| k__Bacteri | Bacteria | Firmicutes | Bacilli | Lactobacilla | Enterococ | Enterococ | dispar | Enterococ |
| k__Bacteri | Bacteria | Firmicutes | Negativicut | Selenomon | Sporomusa | Anaerospo | hongkonge | Anaerospo |
| k__Bacteri | Bacteria | Proteobact | Gammapro | Moraxellal | Moraxellac | Psychroba | urativorans | Psychroba |
| k__Bacteri | Bacteria | Firmicutes | Clostridia | Eubacterial | Clostridiac | Clostridium | cochleariu | Clostridium |
| k__Bacteri | Bacteria | Firmicutes | Bacilli | Bacillales | Bacillaceae | Cytobacillu | ciccensis | Bacillus cic |
| k__Bacteri | Bacteria | Firmicutes | Clostridia | Eubacterial | Lachnospir | Anaerocolu | aminovaler | Anaerocolu |
| k__Bacteri | Bacteria | Proteobact | Gammapro | Enterobact | Enterobact | Siccibacter | turicensis | Siccibacter |
| k__Bacteri | Bacteria | Cyanobact | unclassifie | Nostocales | Hapalosiph | Fischerella | thermalis | Fischerella |
| k__Bacteri | Bacteria | Proteobact | Gammapro | Enterobact | Enterobact | Atlantibact | hermannii | Atlantibact |
| k__Bacteri | Bacteria | Proteobact | Betaprote | Burkholder | Comamona | Variovorax | soli | Variovorax |
| k__Bacteri | Bacteria | Proteobact | Gammapro | Moraxellal | Moraxellac | Acinetobac | schindleri | Acinetobac |
| k__Bacteri | Bacteria | Firmicutes | Bacilli | Bacillales | Bacillaceae | Virgibacillu | dokdonens | Virgibacillu |
| k__Bacteri | Bacteria | Proteobact | Alphaprote | Caulobacte | Caulobacte | Brevundim | halotolera | Brevundim |
| k__Bacteri | Bacteria | Firmicutes | Clostridia | Eubacterial | Clostridiac | Clostridium | huakuii | Clostridium |
| k__Bacteri | Bacteria | Proteobact | Gammapro | Xanthomor | Xanthomor | Stenotroph | terrae | Stenotroph |
| k__Bacteri | Bacteria | Firmicutes | Bacilli | Bacillales | Planococca | Sporosarci | aquimarina | Sporosarci |
| k__Bacteri | Bacteria | Proteobact | Gammapro | Enterobact | Erwiniacea | Pantoea | [Curtobact | [Curtobact |
| k__Bacteri | Bacteria | Firmicutes | Bacilli | Bacillales | Staphyloco | Staphyloco | saccharoly | Staphyloco |
| k__Bacteri | Bacteria | Firmicutes | Bacilli | Bacillales | Bacillaceae | Cytobacillu | eiseniae | Bacillus eis |
| k__Bacteri | Bacteria | Actinobact | Actinomyc | Micrococca | Microbacte | Microbacte | resistens | Microbacte |
| k__Bacteri | Bacteria | Firmicutes | Bacilli | Bacillales | Bacillaceae | Salinibacillu | xinjiangens | Salinibacillu |
| k__Bacteri | Bacteria | Firmicutes | Bacilli | Bacillales | Bacillaceae | Lottiidibaci | patelloidae | Lottiidibaci |
| k__Bacteri | Bacteria | Firmicutes | Bacilli | Bacillales | Planococca | Solibacillus | isronensis | Solibacillus |
| k__Bacteri | Bacteria | Firmicutes | Bacilli | Bacillales | Bacillaceae | Priestia | aryabhatta | Bacillus ary |
| k__Bacteri | Bacteria | Proteobact | Alphaprote | Caulobacte | Caulobacte | Brevundim | vesicularis | Brevundim |
| k__Bacteri | Bacteria | Firmicutes | Bacilli | Bacillales | Bacillaceae | Metabacillu | indicus | Bacillus ind |
| k__Bacteri | Bacteria | Actinobact | Actinomyc | Micrococca | Cellulomor | Oerskovia | turbata | Oerskovia t |
| k__Bacteri | Bacteria | Proteobact | Gammapro | Enterobact | Enterobact | Citrobacter | freundii | Citrobacter |
| k__Bacteri | Bacteria | Actinobact | Actinomyc | Micrococca | Micrococca | Kocuria | assamensis | Kocuria ass |
| k__Bacteri | Bacteria | Firmicutes | Tissierellia | Tissierella | Tissierellac | Tissierella | creatinini | Tissierella |

| | | | | | | | | |
|---|---|---|---|---|---|---|---|---|
| k__Bacteri | Bacteria | Firmicutes | Bacilli | Bacillales | Staphyloco | Staphyloco | ureilyticus | Staphyloco |
| k__Bacteri | Bacteria | Proteobact | Gammapro | Enterobact | Enterobact | Escherichia | fergusonii | Escherichia |
| k__Bacteri | Bacteria | Proteobact | Alphaprote | Hyphomicr | Rhizobiace | Rhizobium | skierniewic | Rhizobium |
| k__Bacteri | Bacteria | Firmicutes | Bacilli | Bacillales | Bacillaceae | Lysinibacill | contaminar | Lysinibacill |
| k__Bacteri | Bacteria | Proteobact | Gammapro | Enterobact | Enterobact | Salmonella | bongori | Salmonella |
| k__Bacteri | Bacteria | Actinobact | Actinomyc | Micrococca | Microbacte | Okibacteriu | fritillariae | Okibacteriu |
| k__Bacteri | Bacteria | Firmicutes | Tissierellia | unclassified | unclassified | Sedimentib | saalensis | Sedimentib |
| k__Bacteri | Bacteria | Bacteroide | Flavobacte | Flavobacte | Schleiferia | Phaeocysti | luteus | Phaeocysti |
| k__Bacteri | Bacteria | Actinobact | Actinomyc | Micrococca | Microbacte | Leucobacte | aridicollis | Leucobacte |
| k__Bacteri | Bacteria | Proteobact | Alphaprote | Hyphomicr | Devosiacea | Devosia | chinhatens | Devosia ch |
| k__Bacteri | Bacteria | Firmicutes | Bacilli | Bacillales | Paenibacill | Paenibacill | etheri | Paenibacill |
| k__Bacteri | Bacteria | Firmicutes | Tissierellia | Tissierellal | Tissierellac | Anaerosali | bizertensis | Anaerosali |
| k__Bacteri | Bacteria | Firmicutes | Bacilli | Bacillales | Bacillaceae | Priestia | flexa | Bacillus fle |
| k__Bacteri | Bacteria | Bacteroide | Flavobacte | Flavobacte | Flavobacte | Flavobacte | rakeshii | Flavobacte |
| k__Bacteri | Bacteria | Actinobact | Actinomyc | Micrococca | Microbacte | Leucobacte | alluvii | Leucobacte |
| k__Bacteri | Bacteria | Firmicutes | Clostridia | Eubacterial | Lachnospir | Lachnoclos | [Clostridiu | [Clostridiu |
| k__Bacteri | Bacteria | Proteobact | Gammapro | Pseudomo | Pseudomo | Pseudomo | aeruginosa | Pseudomo |
| k__Bacteri | Bacteria | Firmicutes | Bacilli | Bacillales | Paenibacill | Paenibacill | thiaminoly | Paenibacill |
| k__Bacteri | Bacteria | Proteobact | Gammapro | Enterobact | Erwiniacea | Pantoea | anthophila | Pantoea an |
| k__Bacteri | Bacteria | Firmicutes | Clostridia | Eubacterial | Clostridiac | Clostridium | sartagofor | Clostridium |
| k__Bacteri | Bacteria | Firmicutes | Bacilli | Bacillales | Bacillaceae | Psychrobac | soli | Psychrobac |
| k__Bacteri | Bacteria | Firmicutes | Bacilli | Bacillales | unclassified | Exiguobact | acetylicum | Exiguobact |
| k__Bacteri | Bacteria | Firmicutes | Bacilli | Bacillales | Planococca | Planomicro | iranicum | Planomicro |
| k__Bacteri | Bacteria | Firmicutes | Bacilli | Bacillales | Bacillaceae | Rossellomo | aquimaris | Bacillus aq |
| k__Bacteri | Bacteria | Firmicutes | Bacilli | Bacillales | Paenibacill | Paenibacill | lutimineral | Paenibacill |
| k__Bacteri | Bacteria | Proteobact | Alphaprote | Sphingomo | Sphingomo | Sphingomo | carotinifaci | Sphingomo |
| k__Bacteri | Bacteria | Firmicutes | Bacilli | Bacillales | Bacillaceae | Cytobacillu | praedii | Bacillus pra |
| k__Bacteri | Bacteria | Firmicutes | Clostridia | Eubacterial | Clostridiac | Clostridium | senegalens | Clostridium |
| k__Bacteri | Bacteria | Actinobact | Actinomyc | Micrococca | Microbacte | Leucobacte | iarius | Leucobacte |
| k__Bacteri | Bacteria | Firmicutes | Clostridia | Eubacterial | unclassified | Natranaero | hydrolytica | Natranaero |
| k__Bacteri | Bacteria | Firmicutes | Bacilli | Bacillales | Planococca | Ureibacillus | manganicu | Lysinibacill |
| k__Bacteri | Bacteria | Proteobact | Gammapro | Enterobact | Erwiniacea | Erwinia | billingiae | Erwinia bill |
| k__Bacteri | Bacteria | Firmicutes | Bacilli | Bacillales | Bacillaceae | Gottfriedia | solisilvae | Bacillus sol |
| k__Bacteri | Bacteria | Firmicutes | Bacilli | Bacillales | unclassified | Exiguobact | himgiriensi | Exiguobact |
| k__Bacteri | Bacteria | Actinobact | Actinomyc | Corynebact | Dietziaceae | Dietzia | cinnamea | Dietzia cinr |
| k__Bacteri | Bacteria | Proteobact | Gammapro | Pseudomo | Pseudomo | Pseudomo | guariconen | Pseudomo |
| k__Bacteri | Bacteria | Firmicutes | Bacilli | Lactobacill | Carnobacte | Atopostipe | suicloacalis | Atopostipe |
| k__Bacteri | Bacteria | Firmicutes | Bacilli | Bacillales | Paenibacill | Paenibacill | purispatii | Paenibacill |
| k__Bacteri | Bacteria | Proteobact | Betaproted | Burkholder | Oxalobacte | Massilia | aquatica | Massilia aq |
| k__Bacteri | Bacteria | Cyanobact | unclassified | Nostocales | Tolypothric | Kryptousia | microlepis | Kryptousia |
| k__Bacteri | Bacteria | Proteobact | Alphaprote | Caulobacte | Caulobacte | Brevundim | staleyi | Brevundim |
| k__Bacteri | Bacteria | Firmicutes | Bacilli | Lactobacill | Streptococ | Lactococcu | lactis | Lactococcu |
| k__Bacteri | Bacteria | Firmicutes | Bacilli | Bacillales | Bacillaceae | Margalitia | camelliae | Bacillus car |
| k__Bacteri | Bacteria | Proteobact | Gammapro | Moraxellal | Moraxellac | Psychrobac | arenosus | Psychrobac |
| k__Bacteri | Bacteria | Proteobact | Alphaprote | Caulobacte | Caulobacte | Brevundim | terrae | Brevundim |
| k__Bacteri | Bacteria | Firmicutes | Bacilli | Bacillales | Bacillaceae | Metabacillu | idriensis | Bacillus idri |
| k__Bacteri | Bacteria | Proteobact | Gammapro | Pseudomo | Pseudomo | Pseudomo | asuensis | Pseudomo |
| k__Bacteri | Bacteria | Actinobact | Actinomyc | Micrococca | Cellulomon | Cellulomon | massiliensi | Cellulomon |
| k__Bacteri | Bacteria | Actinobact | Actinomyc | Micrococca | Dermabact | Brachybact | horti | Brachybact |
| k__Bacteri | Bacteria | Actinobact | Actinomyc | Propioniba | Propioniba | Tessaracoc | rhinoceroti | Tessaracoc |

| | | | | | | | | |
|---|---|---|---|---|---|---|---|---|
| k__Bacteria | Bacteria | Firmicutes | Bacilli | Bacillales | Bacillaceae | Neobacillus | niacini | Bacillus nia |
| k__Bacteria | Bacteria | Proteobact | Alphaprote | Sphingomo | Sphingomo | Sphingomo | adhaesiva | Sphingomo |
| k__Bacteria | Bacteria | Actinobact | Actinomyce | Micrococca | Micrococca | Pseudarthr | scleromae | Pseudarthr |
| k__Bacteria | Bacteria | Proteobact | Betaproteo | Burkholder | Oxalobacte | Massilia | phosphatily | Massilia ph |
| k__Bacteria | Bacteria | Actinobact | Actinomyce | Micrococca | Microbacte | Microbacte | laevaniforn | Microbacte |
| k__Bacteria | Bacteria | Proteobact | Alphaprote | Rhodospiril | Acetobacte | Roseomona | gilardii | Roseomona |
| k__Bacteria | Bacteria | Firmicutes | Bacilli | Lactobacilla | Enterococc | Vagococcus | fluvialis | Vagococcus |
| k__Bacteria | Bacteria | Proteobact | Gammapro | Enterobact | Enterobact | Kluyvera | cryocresce | Kluyvera cr |
| k__Bacteria | Bacteria | Proteobact | Gammapro | Cellvibriona | Cellvibriona | Teredinibac | turnerae | Teredinibac |
| k__Bacteria | Bacteria | Proteobact | Gammapro | Enterobact | Erwiniacea | Pantoea | dispersa | Pantoea dis |
| k__Bacteria | Bacteria | Bacteroide | Bacteroidia | Marinilabili | Marinilabili | Saccharicri | fermentans | Saccharicri |
| k__Bacteria | Bacteria | Proteobact | Gammapro | Xanthomor | Xanthomor | Xanthomor | maliensis | Xanthomor |
| k__Bacteria | Bacteria | Proteobact | Gammapro | Moraxellal | Moraxellac | Acinetobac | junii | Acinetobac |
| k__Bacteria | Bacteria | Firmicutes | Bacilli | Bacillales | Paenibacilla | Paenibacillu | lentimorbu | Paenibacillu |
| k__Bacteria | Bacteria | Firmicutes | Bacilli | Bacillales | Bacillaceae | Cytobacillu | gottheilii | Bacillus got |
| k__Bacteria | Bacteria | Proteobact | Gammapro | Xanthomor | Xanthomor | Stenotroph | koreensis | Stenotroph |
| k__Bacteria | Bacteria | Firmicutes | Bacilli | Bacillales | Bacillaceae | Priestia | qingshengi | Bacillus qin |
| k__Bacteria | Bacteria | Actinobact | Actinomyce | Propioniba | Propioniba | Luteococcu | japonicus | Luteococcu |
| k__Bacteria | Bacteria | Firmicutes | Bacilli | Lactobacilla | Streptococ | Streptococ | thermophil | Streptococ |
| k__Bacteria | Bacteria | Firmicutes | Bacilli | Bacillales | Bacillaceae | Bacillus | aquiflavi | Bacillus aqu |
| k__Bacteria | Bacteria | Cyanobacte | unclassified | Oscillatoria | Microcolea | Tychonema | bourrellyi | Tychonema |
| k__Bacteria | Bacteria | Firmicutes | Clostridia | Eubacterial | Peptococca | Desulfitoba | hafniense | Desulfitoba |
| k__Bacteria | Bacteria | Firmicutes | Bacilli | Bacillales | Bacillaceae | Cytobacillu | oceanisedi | Bacillus oce |
| k__Bacteria | Bacteria | Firmicutes | Bacilli | Bacillales | Bacillaceae | Neobacillus | mesonae | Bacillus me |
| k__Bacteria | Bacteria | Firmicutes | Bacilli | Bacillales | Bacillaceae | Lysinibacillu | parviboron | Lysinibacillu |
| k__Bacteria | Bacteria | Firmicutes | Bacilli | Bacillales | Bacillaceae | Bacillus | dakarensis | Bacillus dak |
| k__Bacteria | Bacteria | Proteobact | Gammapro | Pseudomor | Pseudomor | Pseudomor | floridensis | Pseudomor |
| k__Bacteria | Bacteria | Firmicutes | Bacilli | Bacillales | Bacillaceae | Litchfieldia | alkalitelluri | Bacillus alk |
| k__Bacteria | Bacteria | Firmicutes | Bacilli | Bacillales | Bacillaceae | Ornithiniba | halotolerar | Ornithiniba |
| k__Bacteria | Bacteria | Firmicutes | Bacilli | Bacillales | Paenibacilla | Brevibacillu | laterosporu | Brevibacillu |
| k__Bacteria | Bacteria | Firmicutes | Clostridia | Eubacterial | Eubacteria | Rhabdanae | thermarum | Rhabdanae |
| k__Bacteria | Bacteria | Firmicutes | Bacilli | Bacillales | Bacillaceae | Peribacillus | simplex | Bacillus sim |
| k__Bacteria | Bacteria | Proteobact | Alphaprote | Hyphomicr | Rhizobiacea | Rhizobium | daejeonens | Rhizobium |
| k__Bacteria | Bacteria | Actinobact | Actinomyce | Propioniba | Propioniba | Tessaracoc | lubricantis | Tessaracoc |
| k__Bacteria | Bacteria | Firmicutes | Bacilli | Bacillales | Paenibacilla | Paenibacillu | taohuashar | Paenibacillu |
| k__Bacteria | Bacteria | Firmicutes | Bacilli | Bacillales | Bacillaceae | Metabacillu | endolithicu | Bacillus enc |
| k__Bacteria | Bacteria | Actinobact | Actinomyce | Micrococca | Microbacte | Plantibacte | auratus | Plantibacte |
| k__Bacteria | Bacteria | Firmicutes | Bacilli | Bacillales | Bacillaceae | Bacillus | cecembens | Bacillus cec |
| k__Bacteria | Bacteria | Proteobact | Gammapro | Xanthomor | Xanthomor | Pseudoxan | jiangsuensi | Pseudoxant |
| k__Bacteria | Bacteria | Firmicutes | Clostridia | Eubacterial | Clostridiace | Clostridium | hydrogenif | Clostridium |
| k__Bacteria | Bacteria | Actinobact | Actinomyce | Micrococca | Microbacte | Curtobacte | herbarum | Curtobacte |

| Identity | Sequence | Dongdaemun | Euljiro-1-ga | Sinsa | Yeongdeungpo |
|---|---|---|---|---|---|
| 99.336 | CCTACGGG | 0.63% | 1.80% | 0.62% | 5.00% |
| 99.333 | GCCTACGG | 0.10% | 0.39% | 1.28% | 1.42% |
| 99.336 | CCTACGGG | 3.35% | 42.32% | 4.54% | 1.71% |
| 99.336 | CCTACGGG | 0.40% | 0.01% | 0.02% | 0.13% |
| 99.336 | CCTACGGG | 0.56% | 0.01% | 0.00% | 0.01% |
| 99.336 | TCCTACGG | 1.25% | 0.00% | 22.90% | 7.26% |
| 99.336 | CCTACGGG | 1.81% | 0.85% | 0.33% | 0.05% |
| 99.219 | GACTACTG | 0.25% | 0.60% | 0.24% | 1.54% |
| 95.364 | CCTACGGG | 13.98% | 0.00% | 5.85% | 3.37% |
| 99.336 | CCTACGGG | 0.55% | 3.48% | 6.12% | 14.57% |
| 100 | GACTACCA | 0.52% | 0.00% | 0.07% | 0.40% |
| 99.336 | CCTACGGG | 0.00% | 0.00% | 0.01% | 0.00% |
| 99.336 | CCTACGGG | 0.00% | 0.01% | 0.00% | 0.01% |
| 99.333 | GCCTACGG | 12.89% | 0.01% | 0.00% | 0.05% |
| 99.336 | CCTACGGG | 0.00% | 0.00% | 0.00% | 0.14% |
| 95.681 | CCTACGGG | 0.07% | 0.00% | 0.90% | 1.65% |
| 99.336 | CCTACGGG | 18.66% | 0.01% | 0.19% | 2.70% |
| 99.609 | GACTACCG | 9.61% | 0.01% | 0.01% | 0.04% |
| 99.336 | CCTACGGG | 0.00% | 0.00% | 0.00% | 0.00% |
| 99.336 | CCTACGGG | 0.07% | 0.00% | 1.25% | 0.39% |
| 98.339 | CCTACGGG | 0.23% | 0.00% | 0.83% | 0.01% |
| 99.336 | CCTACGGG | 0.62% | 0.04% | 1.55% | 4.08% |
| 99.336 | CCTACGGG | 0.00% | 0.00% | 0.00% | 0.00% |
| 99 | GCCTACGG | 14.38% | 0.00% | 0.01% | 0.46% |
| 98.828 | GACTACAG | 0.01% | 0.08% | 0.00% | 0.00% |
| 99.336 | CCTACGGG | 0.00% | 0.00% | 0.01% | 0.13% |
| 98.007 | CCTACGGG | 0.00% | 0.00% | 0.00% | 0.00% |
| 95.667 | GCCTACGG | 1.62% | 0.00% | 13.35% | 0.16% |
| 99.003 | CCTACGGG | 0.00% | 0.00% | 0.00% | 0.00% |
| 97.342 | CCTACGGG | 0.02% | 0.01% | 0.05% | 1.45% |
| 99.336 | CCTACGGG | 0.00% | 0.00% | 0.00% | 0.00% |
| 99.003 | CCTACGGG | 0.13% | 0.00% | 2.38% | 0.73% |
| 99.333 | GCCTACGG | 0.00% | 0.00% | 0.00% | 0.12% |
| 99.119 | GACTACAG | 0.54% | 6.27% | 0.84% | 0.65% |
| 99.003 | CCTACGGG | 0.14% | 12.01% | 0.57% | 0.13% |
| 99.336 | CCTACGGG | 0.19% | 0.00% | 0.83% | 12.45% |
| 99.115 | GGACTACT | 0.00% | 1.61% | 0.03% | 0.62% |
| 99.336 | CCTACGGG | 0.18% | 0.00% | 0.88% | 1.05% |
| 98.671 | CCTACGGG | 0.19% | 0.00% | 0.45% | 10.25% |
| 99.336 | CCTACGGG | 0.22% | 0.00% | 0.03% | 0.00% |
| 99.003 | CCTACGGG | 0.08% | 0.31% | 1.66% | 4.80% |
| 100 | GACTACCA | 0.01% | 1.32% | 0.07% | 0.58% |
| 99.329 | CCTACGGG | 0.00% | 0.00% | 0.00% | 0.00% |
| 99.336 | CCTACGGG | 2.46% | 7.29% | 0.00% | 0.02% |
| 99.336 | CCTACGGG | 0.13% | 8.29% | 0.01% | 0.01% |
| 83.122 | CCTACGGG | 0.00% | 0.02% | 9.20% | 0.00% |
| 99.585 | GACTACCG | 2.27% | 5.30% | 0.06% | 0.03% |
| 98.339 | CCTACGGG | 0.00% | 0.00% | 0.01% | 4.30% |
| 99.219 | GACTACAG | 0.00% | 0.00% | 0.00% | 0.00% |

| | | | | | | |
|---|---|---|---|---|---|---|
| ✓ | 98.671 | CCTACGGG | 0.00% | 0.00% | 0.00% | 0.00% |
| ✓ | 99.327 | TTCCTACGG | 0.00% | 0.00% | 0.00% | 0.01% |
| ✓ | 99.003 | CCTACGGG | 0.01% | 0.00% | 0.04% | 0.04% |
| ✓ | 99.336 | CCTACGGG | 0.00% | 0.00% | 0.00% | 0.00% |
| ✓ | 99.336 | CCTACGGG | 0.00% | 0.00% | 0.00% | 0.00% |
| ✓ | 99.333 | GCCTACGG | 0.15% | 0.00% | 0.04% | 0.01% |
| ✓ | 100 | GACTACCA | 0.00% | 0.00% | 0.01% | 0.06% |
| ✓ | 99.336 | CCTACGGG | 0.00% | 0.00% | 0.00% | 0.00% |
| ✓ | 99.336 | CCTACGGG | 0.00% | 0.00% | 0.00% | 0.00% |
| ✓ | 99.6 | GACTACCG | 0.01% | 0.54% | 0.16% | 0.05% |
| ✓ | 99.336 | CCTACGGG | 0.04% | 0.21% | 0.07% | 0.10% |
| ✓ | 99 | GCCTACGG | 0.00% | 0.00% | 0.00% | 0.00% |
| ✓ | 99.336 | CCTACGGG | 0.00% | 0.00% | 0.00% | 0.00% |
| ✓ | 99.336 | CCTACGGG | 0.00% | 0.44% | 0.00% | 1.03% |
| ✗ | 82.508 | CCTACGGG | 0.00% | 0.00% | 5.03% | 0.94% |
| ✓ | 99.336 | CCTACGGG | 0.01% | 0.00% | 0.01% | 0.18% |
| ✓ | 98.671 | CCTACGGG | 0.01% | 0.00% | 0.00% | 0.00% |
| ✓ | 98.671 | CCTACGGG | 0.01% | 0.01% | 0.02% | 0.06% |
| ✓ | 99.336 | CCTACGGG | 0.00% | 0.00% | 0.00% | 0.00% |
| ✓ | 99 | TTCCTACGG | 0.00% | 0.00% | 0.00% | 0.00% |
| ✓ | 99.003 | CCTACGGG | 0.00% | 0.02% | 0.01% | 0.02% |
| ✓ | 99.336 | CCTACGGG | 0.00% | 0.00% | 0.01% | 0.00% |
| ✓ | 99.336 | CCTACGGG | 0.00% | 0.00% | 0.04% | 0.23% |
| ✓ | 99.216 | GACTACAG | 0.25% | 0.00% | 0.00% | 0.00% |
| ✓ | 99.003 | CCTACGGG | 0.04% | 1.64% | 0.13% | 2.73% |
| ✓ | 99.333 | GCCTACGG | 0.01% | 0.20% | 0.01% | 0.03% |
| ✓ | 99.336 | CCTACGGG | 0.00% | 0.00% | 0.00% | 0.00% |
| ✓ | 99.558 | GACTACCG | 0.00% | 0.00% | 0.00% | 0.00% |
| ✓ | 99.331 | GCCTACGG | 0.01% | 0.30% | 0.25% | 0.75% |
| ✓ | 99.115 | GACTACAG | 0.01% | 0.00% | 0.19% | 0.06% |
| ✓ | 99.559 | GACTACAA | 0.13% | 0.04% | 2.39% | 0.04% |
| ✓ | 98.339 | CCTACGGG | 0.03% | 0.00% | 0.19% | 0.06% |
| ✓ | 99.336 | CCTACGGG | 0.04% | 0.00% | 0.00% | 0.00% |
| ✓ | 98.734 | CCTACGGG | 0.02% | 0.00% | 0.21% | 0.00% |
| ✓ | 99.003 | CCTACGGG | 0.00% | 0.00% | 0.00% | 0.00% |
| ✓ | 99.336 | CCTACGGG | 2.58% | 0.00% | 0.00% | 0.15% |
| ✓ | 99.336 | CCTACGGG | 0.18% | 0.00% | 0.00% | 0.00% |
| ✓ | 99.559 | GACTACTA | 0.00% | 0.00% | 0.01% | 0.00% |
| ✓ | 100 | GACTACCA | 0.00% | 0.00% | 0.00% | 0.03% |
| ✓ | 99.331 | TACCTACGG | 0.00% | 1.89% | 0.04% | 0.11% |
| ✓ | 99.336 | CCTACGGG | 0.00% | 0.00% | 0.00% | 0.00% |
| ✓ | 99 | GCCTACGG | 0.02% | 0.14% | 0.31% | 0.03% |
| ✓ | 98.662 | TACCTACGG | 0.01% | 0.00% | 0.05% | 0.01% |
| ✓ | 99.336 | CCTACGGG | 2.57% | 0.00% | 0.00% | 0.01% |
| ✓ | 99.17 | GACTACAG | 0.00% | 0.00% | 0.00% | 0.00% |
| ✓ | 99.559 | GACTACCG | 0.03% | 0.01% | 0.10% | 1.91% |
| ✓ | 98.339 | CCTACGGG | 0.00% | 0.00% | 0.00% | 0.00% |
| ✓ | 98.007 | CCTACGGG | 0.01% | 0.00% | 0.54% | 0.00% |
| ✓ | 98 | CCTACGGG | 0.00% | 0.00% | 0.01% | 0.29% |
| ✓ | 99.219 | GACTACAG | 0.03% | 0.00% | 0.00% | 0.00% |

| | | | | | | |
|---|---|---|---|---|---|---|
| | 86.222 | GCCTACGG | 0.01% | 0.02% | 1.92% | 0.39% |
| | 98.222 | TGACTACC | 1.70% | 0.30% | 0.00% | 0.05% |
| | 99.119 | GACTACAG | 0.00% | 0.00% | 0.01% | 0.01% |
| | 99.2 | GACTACAG | 0.00% | 0.00% | 0.00% | 0.00% |
| | 99.336 | CCTACGGG | 0.07% | 0.00% | 2.38% | 0.00% |
| | 96.346 | CCTACGGG | 0.00% | 0.00% | 0.00% | 0.01% |
| | 99.003 | CCTACGGG | 0.00% | 0.11% | 0.02% | 0.58% |
| | 99.336 | CCTACGGG | 0.00% | 0.00% | 2.21% | 0.00% |
| | 97.5 | CCTACGGG | 0.00% | 0.00% | 0.00% | 0.00% |
| | 99.667 | TTCCTACG | 0.00% | 0.03% | 0.01% | 0.00% |
| | 97.674 | CCTACGGG | 0.00% | 0.02% | 0.00% | 0.00% |
| | 99.119 | TGACTACT | 0.00% | 0.00% | 0.07% | 0.02% |
| | 99.6 | GACTACCG | 0.00% | 0.00% | 0.00% | 0.00% |
| | 99.336 | CCTACGGG | 1.58% | 0.00% | 0.00% | 0.07% |
| | 87.234 | CCTACGGG | 0.02% | 0.04% | 0.51% | 0.46% |
| | 96.013 | CCTACGGG | 0.00% | 0.00% | 0.00% | 0.02% |
| | 99.336 | CCTACGGG | 0.16% | 0.17% | 1.01% | 0.11% |
| | 83.974 | GGACTACC | 0.00% | 0.00% | 1.36% | 0.16% |
| | 95.849 | GACTACAG | 0.00% | 0.00% | 0.01% | 0.25% |
| | 99.336 | CCTACGGG | 0.00% | 0.01% | 0.00% | 0.00% |
| | 100 | GACTACCA | 0.00% | 0.00% | 0.00% | 0.00% |
| | 99.336 | CCTACGGG | 0.00% | 0.00% | 0.01% | 1.41% |
| | 99.336 | CCTACGGG | 0.00% | 0.00% | 0.00% | 0.00% |
| | 98.339 | CCTACGGG | 0.00% | 0.00% | 0.00% | 0.00% |
| | 99.248 | GACTACCG | 0.03% | 0.01% | 0.14% | 0.89% |
| | 99.336 | CCTACGGG | 0.00% | 0.00% | 0.17% | 0.00% |
| | 98.561 | CCTACGGG | 0.00% | 0.00% | 0.00% | 0.01% |
| | 86.735 | CCTACGGG | 0.00% | 0.00% | 0.04% | 0.00% |
| | 99.333 | GCCTACGG | 0.00% | 0.26% | 0.04% | 0.01% |
| | 99.251 | TGACTACA | 0.00% | 0.00% | 0.00% | 0.00% |
| | 99.003 | CCTACGGG | 0.00% | 0.36% | 0.03% | 0.01% |
| | 99.203 | GACTACAG | 0.03% | 0.01% | 0.00% | 0.00% |
| | 99.336 | CCTACGGG | 0.02% | 0.00% | 0.00% | 0.00% |
| | 97.112 | GACTACCG | 0.00% | 0.00% | 0.00% | 0.00% |
| | 99.219 | GACTACAG | 0.00% | 0.00% | 0.02% | 0.00% |
| | 99.331 | GCCTACGG | 0.06% | 0.03% | 0.00% | 0.05% |
| | 98.339 | CCTACGGG | 0.00% | 0.01% | 0.00% | 0.00% |
| | 99.315 | GCCTACGG | 0.00% | 0.00% | 0.00% | 0.00% |
| | 99.336 | CCTACGGG | 0.01% | 0.02% | 0.24% | 0.65% |
| | 99.003 | CCTACGGG | 0.00% | 0.00% | 0.00% | 0.00% |
| | 97.887 | CCTACGGG | 0.02% | 0.01% | 0.04% | 0.01% |
| | 99.336 | CCTACGGG | 0.43% | 0.00% | 0.21% | 0.14% |
| | 98.339 | CCTACGGG | 0.00% | 0.00% | 0.01% | 0.00% |
| | 99.336 | TCCTACGG | 0.00% | 0.00% | 0.02% | 0.01% |
| | 99.259 | GACTACAG | 0.00% | 0.00% | 0.00% | 0.00% |
| | 98.12 | GACTACAG | 0.00% | 0.00% | 0.01% | 0.02% |
| | 99.115 | TGACTACA | 0.00% | 0.00% | 0.00% | 0.00% |
| | 99.003 | CCTACGGG | 0.00% | 0.00% | 0.00% | 0.00% |
| | 99.119 | GACTACAG | 0.00% | 0.00% | 0.00% | 0.00% |
| | 94.352 | CCTACGGG | 0.02% | 0.00% | 0.15% | 0.00% |

| | | | | | |
|---|---|---|---|---|---|
| 99.336 | CCTACGGG | 0.00% | 0.00% | 0.00% | 0.00% |
| 99.336 | CCTACGGG | 0.00% | 0.01% | 0.00% | 0.03% |
| 99.609 | GACTACCG | 0.02% | 0.00% | 0.00% | 0.00% |
| 98.658 | TTCCCTAC | 0.00% | 0.01% | 0.02% | 0.00% |
| 99.2 | GACTACAG | 0.00% | 0.00% | 0.05% | 0.02% |
| 99.336 | CCTACGGG | 0.06% | 0.00% | 0.00% | 0.01% |
| 95.724 | CCTACGGG | 0.00% | 0.00% | 0.00% | 0.00% |
| 92.963 | GACTACAA | 0.00% | 0.00% | 0.03% | 0.00% |
| 99.336 | CCTACGGG | 0.00% | 0.00% | 0.00% | 0.00% |
| 99 | GCCTACGG | 0.32% | 0.00% | 0.00% | 0.10% |
| 99.336 | CCTACGGG | 0.00% | 0.00% | 0.00% | 0.00% |
| 99.336 | CCTACGGG | 0.00% | 0.00% | 0.00% | 0.52% |
| 99.336 | CCTACGGG | 0.00% | 0.00% | 0.00% | 0.00% |
| 92.667 | CCTACGGG | 0.01% | 0.00% | 0.02% | 0.00% |
| 99.18 | GCCTACGG | 0.21% | 0.00% | 0.00% | 0.00% |
| 98.438 | GACTACCA | 0.00% | 0.00% | 0.51% | 0.00% |
| 99.13 | CCTACGGG | 0.01% | 0.00% | 0.27% | 0.07% |
| 99.194 | CCTACGGG | 0.11% | 0.34% | 0.00% | 0.00% |
| 98.997 | TTTCCTAC | 0.00% | 0.00% | 0.01% | 0.29% |
| 99.336 | CCTACGGG | 0.01% | 0.00% | 0.13% | 0.00% |
| 99.609 | GACTACAA | 0.01% | 0.00% | 0.07% | 0.12% |
| 99.336 | CCTACGGG | 0.00% | 0.00% | 0.00% | 0.00% |
| 98 | GACTACTG | 0.01% | 0.07% | 0.01% | 0.02% |
| 99.231 | CCTACGGG | 0.01% | 0.00% | 0.09% | 0.27% |
| 99.245 | GACTACCC | 0.00% | 0.00% | 0.02% | 0.00% |
| 99.329 | TGCCCTAC | 0.00% | 0.00% | 0.00% | 0.00% |
| 98.671 | CCTACGGG | 0.00% | 0.00% | 0.04% | 0.36% |
| 99.336 | CCTACGGG | 0.00% | 0.00% | 0.00% | 0.00% |
| 99.275 | CCTACGGG | 0.13% | 0.00% | 0.00% | 0.00% |
| 90.132 | CCTACGGG | 0.00% | 0.00% | 0.00% | 0.00% |
| 98.007 | CCTACGGG | 0.00% | 0.04% | 0.02% | 0.00% |
| 99.2 | GACTACAG | 0.00% | 0.00% | 0.00% | 0.00% |
| 97.345 | GACTACAG | 0.01% | 0.00% | 0.04% | 0.13% |
| 99.336 | CCTACGGG | 0.01% | 0.00% | 0.36% | 0.00% |
| 99.336 | CCTACGGG | 0.00% | 0.00% | 0.00% | 0.00% |
| 95.279 | TGACTACT | 0.01% | 0.00% | 0.19% | 0.03% |
| 98.997 | TACCTACG | 0.00% | 0.00% | 0.00% | 0.34% |
| 99.336 | CCTACGGG | 0.00% | 0.00% | 0.00% | 0.00% |
| 97.683 | CCCTACGG | 0.00% | 0.00% | 0.00% | 0.00% |
| 88.294 | CCTACGGG | 0.00% | 0.00% | 0.01% | 0.00% |
| 99.565 | CCTACGGG | 0.04% | 0.00% | 0.00% | 0.00% |
| 99.336 | CCTACGGG | 0.00% | 0.00% | 0.00% | 0.00% |
| 97.656 | TGACTACT | 0.00% | 0.00% | 0.00% | 0.00% |
| 99.336 | CCTACGGG | 0.00% | 0.00% | 0.00% | 0.00% |
| 99.13 | CCTACGGG | 0.11% | 0.00% | 0.00% | 0.01% |
| 98.084 | CCTACGGG | 0.00% | 0.00% | 0.00% | 0.00% |
| 100 | GACTACCA | 0.00% | 0.00% | 0.01% | 0.19% |
| 99.336 | CCTACGGG | 0.00% | 0.00% | 0.01% | 0.00% |
| 99.336 | CCTACGGG | 0.00% | 0.19% | 0.00% | 0.00% |
| 99.668 | CCTACGGG | 0.00% | 0.00% | 0.00% | 0.00% |

| | | | | | |
|---|---|---|---|---|---|
| ✓ 99.333 | GACTACTG | 0.01% | 0.00% | 0.02% | 0.10% |
| ✓ 99.127 | CCTACGGG | 0.00% | 0.00% | 0.00% | 0.00% |
| ✓ 97.674 | CCTACGGG | 0.00% | 0.00% | 0.00% | 0.00% |
| ! 96.99 | CCTACGGG | 0.00% | 0.00% | 0.00% | 0.00% |
| ✓ 99.153 | GCCTACGG | 0.00% | 0.00% | 0.00% | 0.00% |
| ✓ 99.668 | CCTACGGG | 0.00% | 0.09% | 0.00% | 0.00% |
| ✓ 99.336 | CCTACGGG | 0.00% | 0.00% | 0.00% | 0.00% |
| ✓ 98.007 | CCTACGGG | 0.00% | 0.00% | 0.00% | 0.00% |
| ! 95.113 | TGACTACT | 0.00% | 0.00% | 0.01% | 0.00% |
| ✓ 98.662 | CCTACGGG | 0.00% | 0.00% | 0.01% | 0.15% |
| ! 92.527 | TCCTACGG | 0.00% | 0.00% | 0.02% | 0.00% |
| ✓ 98.339 | CCTACGGG | 0.00% | 0.00% | 0.00% | 0.00% |
| ✓ 99.336 | CCTACGGG | 0.00% | 0.04% | 0.00% | 0.00% |
| ✓ 99.623 | GACTACCG | 0.04% | 0.12% | 0.00% | 0.00% |
| ✓ 98.667 | GCCTACGG | 0.00% | 0.04% | 0.07% | 0.01% |
| ✓ 98.535 | CCTACGGG | 0.00% | 0.00% | 0.01% | 0.00% |
| ✓ 99.203 | GACTACTG | 0.00% | 0.00% | 0.01% | 0.00% |
| ! 95.017 | CCTACGGG | 0.00% | 0.00% | 0.00% | 0.00% |
| ✓ 99.336 | CCTACGGG | 0.01% | 0.01% | 0.01% | 0.01% |
| ✓ 97.674 | CCTACGGG | 0.10% | 0.03% | 0.01% | 0.01% |
| ✗ 86.842 | CCTACGGG | 0.00% | 0.00% | 0.00% | 0.00% |
| ✓ 100 | TCCTACGG | 0.00% | 0.00% | 0.06% | 0.00% |
| ✓ 99 | TTCCTACG | 0.01% | 0.00% | 0.03% | 0.06% |
| ✓ 99.119 | TTGGACTA | 0.00% | 0.01% | 0.04% | 0.00% |
| ✓ 99.611 | GACTACAA | 0.00% | 0.01% | 0.01% | 0.05% |
| ✓ 99.156 | TGCCTACG | 0.00% | 0.02% | 0.00% | 0.00% |
| ✓ 99.119 | GACTACAC | 0.00% | 0.00% | 0.00% | 0.00% |
| ✓ 98.238 | GGACTACT | 0.00% | 0.00% | 0.00% | 0.07% |
| ✓ 99.115 | TGGGACTA | 0.03% | 0.02% | 0.05% | 0.01% |
| ✓ 97.992 | GACTACAG | 0.00% | 0.00% | 0.00% | 0.00% |
| ! 91.362 | CCTACGGG | 0.00% | 0.00% | 0.00% | 0.11% |
| ✓ 99.219 | GACTACAA | 0.00% | 0.01% | 0.01% | 0.04% |
| ✓ 99.609 | TGACTACC | 0.08% | 0.00% | 0.00% | 0.01% |
| ✓ 97.674 | CCTACGGG | 0.00% | 0.01% | 0.00% | 0.00% |
| ✓ 99.336 | CCTACGGG | 0.00% | 0.00% | 0.00% | 0.00% |
| ✓ 98.238 | GACTACAG | 0.00% | 0.00% | 0.00% | 0.00% |
| ✓ 99.558 | GACTACCA | 0.00% | 0.00% | 0.00% | 0.00% |
| ✓ 99 | GCCTACGG | 0.00% | 0.00% | 0.00% | 0.00% |
| ✓ 98.75 | TTCCTACG | 0.00% | 0.00% | 0.00% | 0.01% |
| ✓ 99.248 | GACTACTG | 0.00% | 0.00% | 0.01% | 0.00% |
| ✓ 100 | GACTACCA | 0.00% | 0.00% | 0.00% | 0.00% |

| Yongsan |
| --- |
| 4.39% |
| 15.90% |
| 1.47% |
| 0.15% |
| 0.02% |
| 8.92% |
| 0.07% |
| 1.53% |
| 10.11% |
| 5.97% |
| 0.07% |
| 0.00% |
| 0.07% |
| 0.35% |
| 2.62% |
| 3.70% |
| 0.41% |
| 0.27% |
| 0.00% |
| 1.18% |
| 0.06% |
| 0.60% |
| 0.00% |
| 0.72% |
| 0.00% |
| 0.08% |
| 0.00% |
| 0.04% |
| 0.00% |
| 0.39% |
| 0.00% |
| 2.86% |
| 0.02% |
| 0.21% |
| 0.02% |
| 0.28% |
| 0.71% |
| 0.02% |
| 0.38% |
| 0.00% |
| 3.92% |
| 1.32% |
| 0.00% |
| 0.03% |
| 0.00% |
| 0.05% |
| 0.06% |
| 0.11% |
| 0.00% |

| |
|---|
| 0.70% |
| 0.01% |
| 0.00% |
| 0.00% |
| 0.02% |
| 0.01% |
| 0.63% |
| 0.00% |
| 0.00% |
| 5.70% |
| 0.04% |
| 0.00% |
| 0.00% |
| 0.65% |
| 0.25% |
| 0.00% |
| 0.00% |
| 5.99% |
| 0.33% |
| 0.00% |
| 0.00% |
| 0.00% |
| 0.03% |
| 0.00% |
| 0.45% |
| 0.00% |
| 0.00% |
| 0.00% |
| 3.36% |
| 0.14% |
| 1.34% |
| 0.00% |
| 0.00% |
| 0.02% |
| 0.00% |
| 0.13% |
| 0.00% |
| 0.02% |
| 0.01% |
| 0.32% |
| 0.01% |
| 0.05% |
| 0.00% |
| 0.03% |
| 0.07% |
| 0.34% |
| 3.00% |
| 0.00% |
| 0.00% |
| 0.00% |

| |
|---|
| 0.06% |
| 0.01% |
| 0.02% |
| 0.31% |
| 0.00% |
| 2.36% |
| 0.51% |
| 0.00% |
| 0.00% |
| 0.00% |
| 0.01% |
| 0.09% |
| 0.00% |
| 0.13% |
| 0.26% |
| 0.00% |
| 0.00% |
| 0.02% |
| 0.00% |
| 0.01% |
| 0.00% |
| 0.00% |
| 0.00% |
| 0.11% |
| 0.02% |
| 0.00% |
| 0.01% |
| 0.00% |
| 0.81% |
| 0.00% |
| 0.00% |
| 0.00% |
| 0.00% |
| 0.04% |
| 0.00% |
| 0.00% |
| 0.00% |
| 0.00% |
| 0.12% |
| 0.00% |
| 0.04% |
| 0.12% |
| 0.08% |
| 0.02% |
| 0.00% |
| 0.00% |
| 0.02% |
| 0.09% |
| 0.00% |
| 0.00% |

| |
|---|
| 0.00% |
| 0.02% |
| 0.00% |
| 0.02% |
| 0.50% |
| 0.00% |
| 0.00% |
| 0.00% |
| 0.00% |
| 0.02% |
| 0.01% |
| 0.00% |
| 0.00% |
| 0.00% |
| 0.00% |
| 0.00% |
| 0.10% |
| 0.00% |
| 0.11% |
| 0.00% |
| 0.00% |
| 0.14% |
| 0.01% |
| 0.01% |
| 0.08% |
| 0.00% |
| 0.00% |
| 0.00% |
| 0.00% |
| 0.00% |
| 0.01% |
| 0.00% |
| 0.00% |
| 0.00% |
| 0.00% |
| 0.04% |
| 0.00% |
| 0.06% |
| 0.00% |
| 0.00% |
| 0.00% |
| 0.00% |
| 0.00% |
| 0.00% |
| 0.01% |
| 0.00% |
| 0.01% |
| 0.07% |
| 0.00% |
| 0.00% |

| |
|---|
| 0.00% |
| 0.00% |
| 0.00% |
| 0.00% |
| 0.00% |
| 0.00% |
| 0.00% |
| 0.18% |
| 0.00% |
| 0.02% |
| 0.00% |
| 0.00% |
| 0.00% |
| 0.00% |
| 0.03% |
| 0.00% |
| 0.00% |
| 0.01% |
| 0.01% |
| 0.00% |
| 0.09% |
| 0.00% |
| 0.02% |
| 0.00% |
| 0.04% |
| 0.11% |
| 0.02% |
| 0.00% |
| 0.01% |
| 0.11% |
| 0.00% |
| 0.00% |
| 0.00% |
| 0.00% |
| 0.00% |
| 0.00% |
| 0.00% |
| 0.03% |
| 0.00% |
| 0.00% |
| 0.00% |
