# Supplementary material for: The composition of the bacterial communities collected from the PM10 samples inside the Seoul subway and railway station
Source: Sci Rep. 2024 Mar 18;14:6478. doi: 10.1038/s41598-023-49848-x (PMC10948816; doi:10.1038/s41598-023-49848-x)
Supplement: Supplementary file 2 — Supplementary Information 2. [file 41598_2023_49848_MOESM2_ESM.docx]

**The composition of the bacterial communities collected from the PM_10_ samples inside the Seoul subway and railway station**

Shambhavi Sharma^1,2^, Muhammad Jahanzaib^1,2^, Ahtesham Bakht^3^, Min-Kyung Kim^1^, Hyunsoo Lee^3^, and Duckshin Park ^1,2*^

^1^Department of Transportation Environmental Research, Korea Railroad Research Institute (KRRI), Uiwang, 16105, South Korea.

^2^Transportation System Engineering, University of Science and Technology (UST), Daejeon, South Korea.

^3^Kumoh National Institute of Technology (KIT), 61 Daehak-ro, Gumi-si, Gyeongsanbuk-do (39177), Republic of Korea.

*Correspondence: [dspark@krri.re.kr](mailto:dspark@krri.re.kr) Tel.: +82-10-3343-2862

## **Supplementary figures**


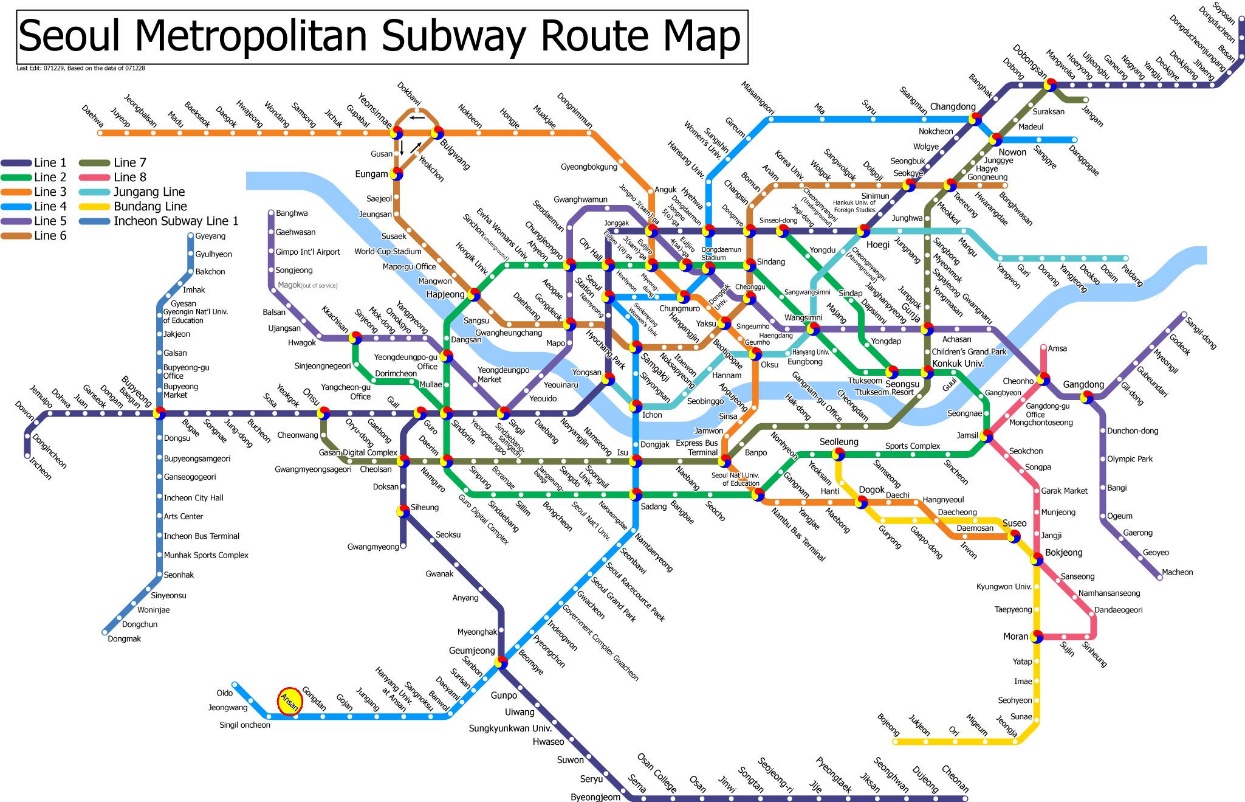


**Fig S1-** The map of Seoul Subway


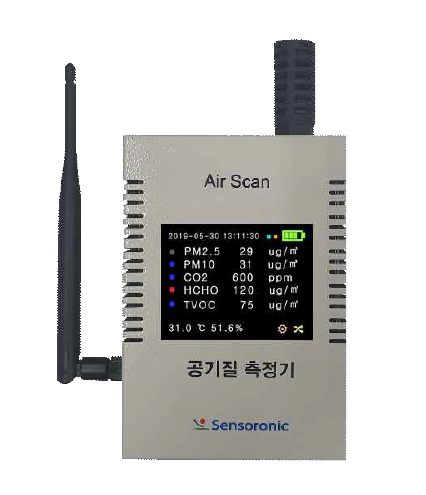


**Fig S2-** Air Scan device


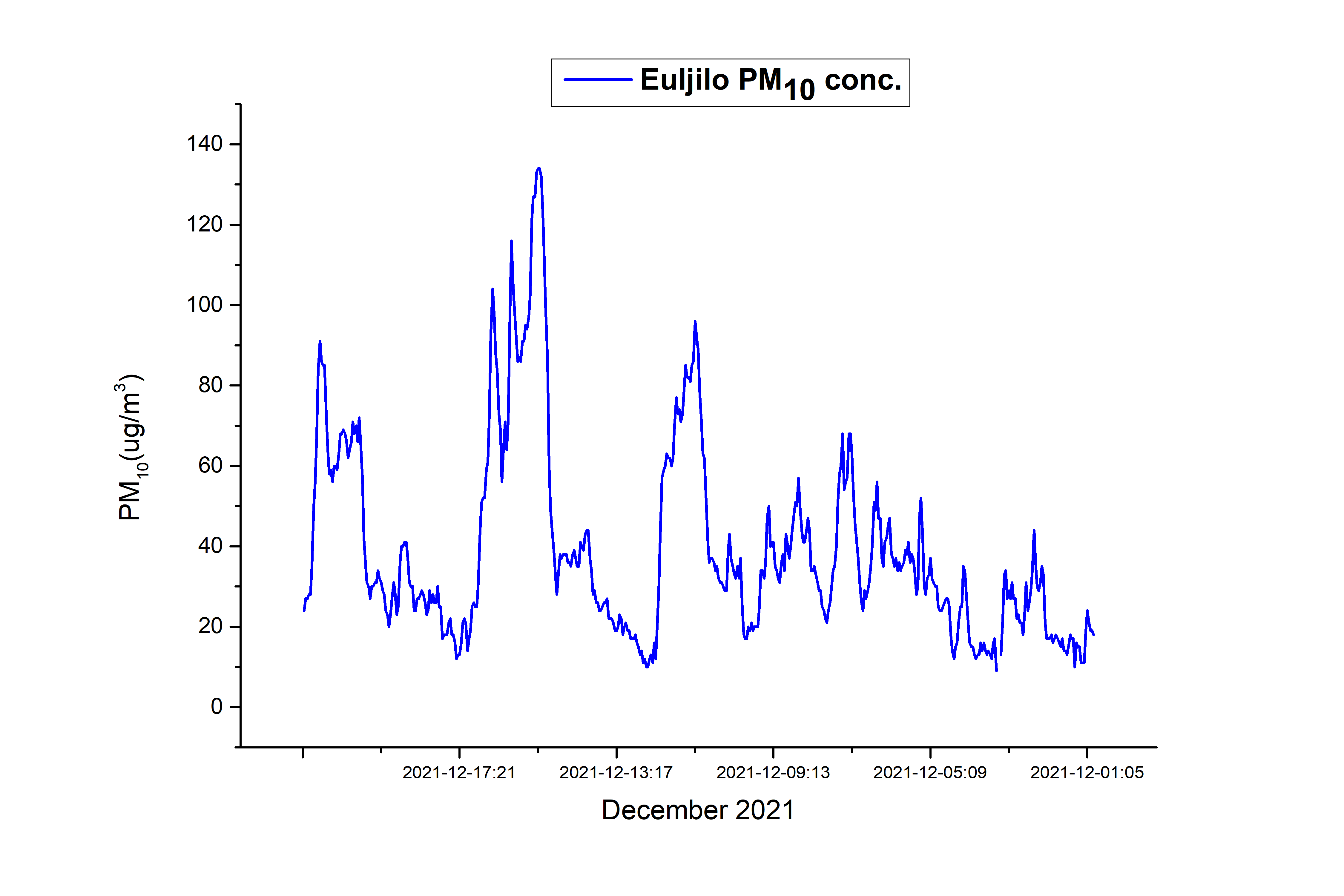


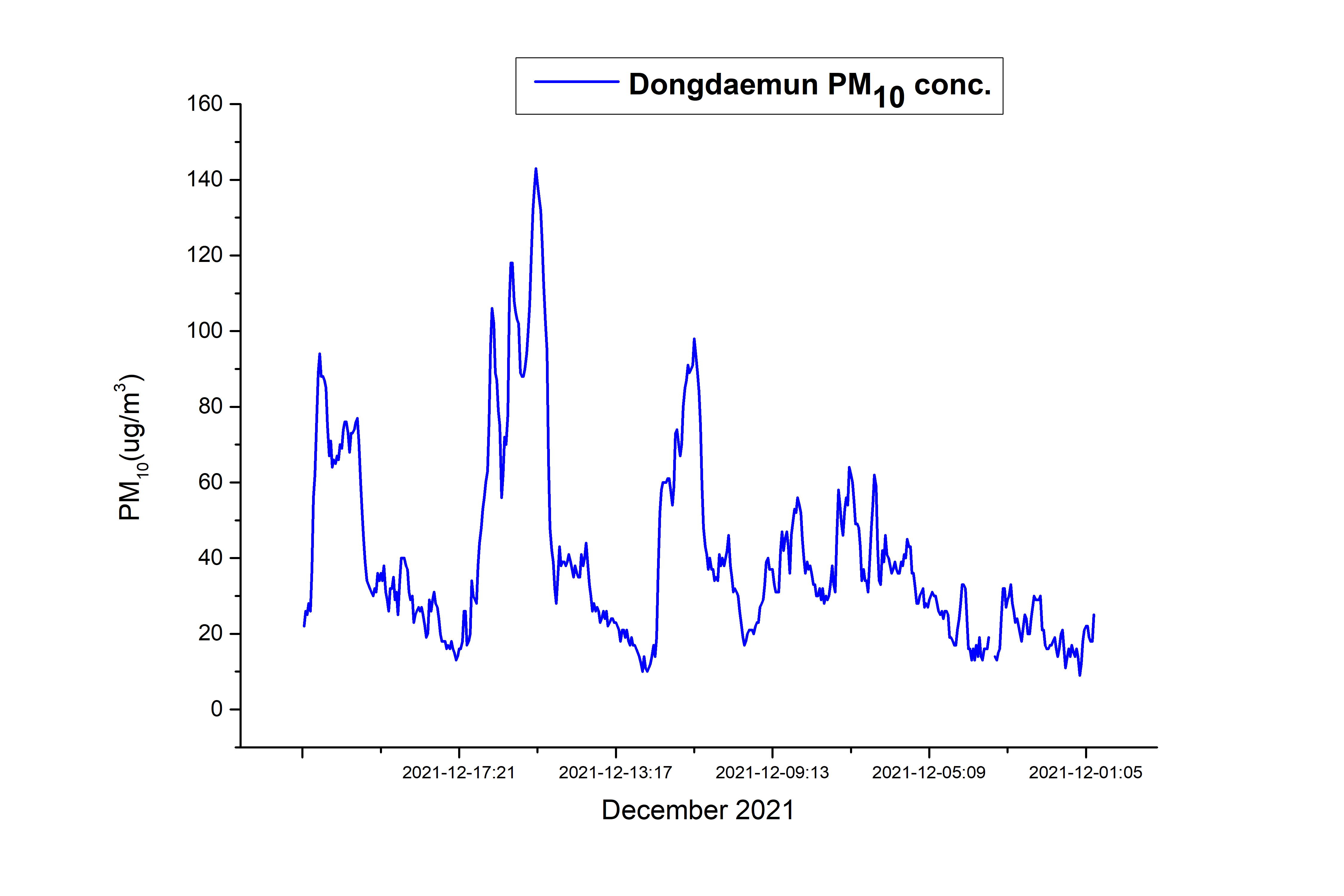


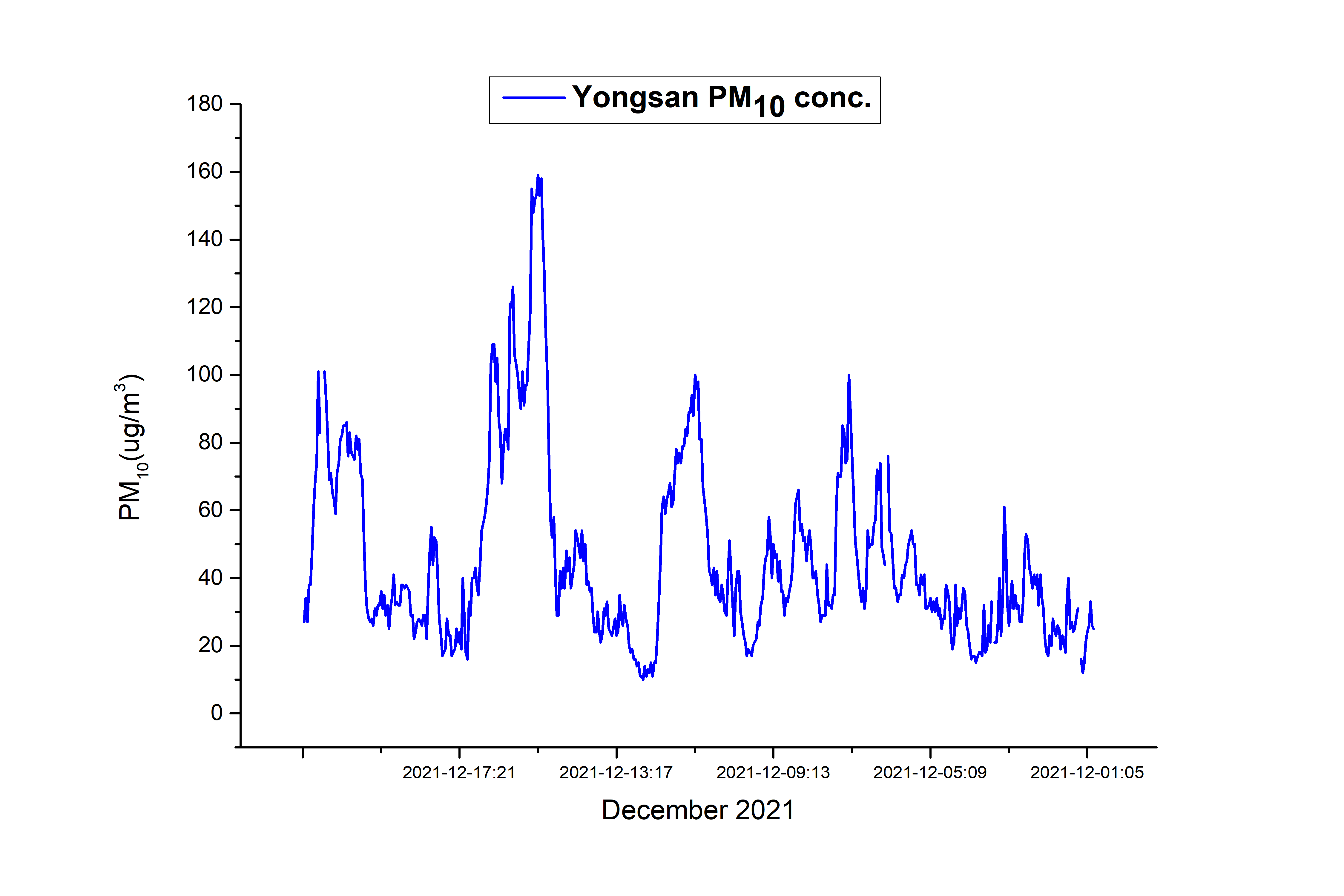


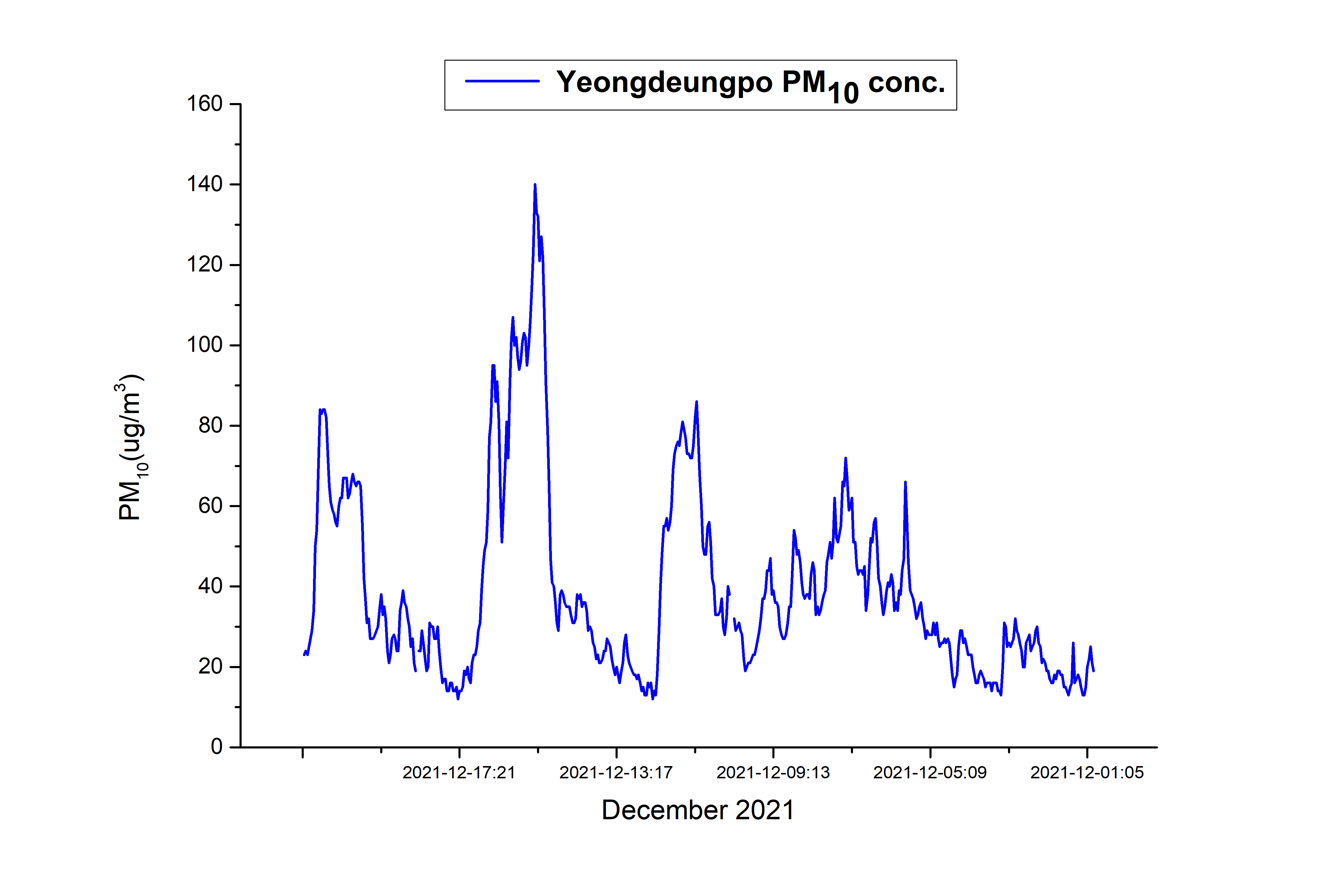


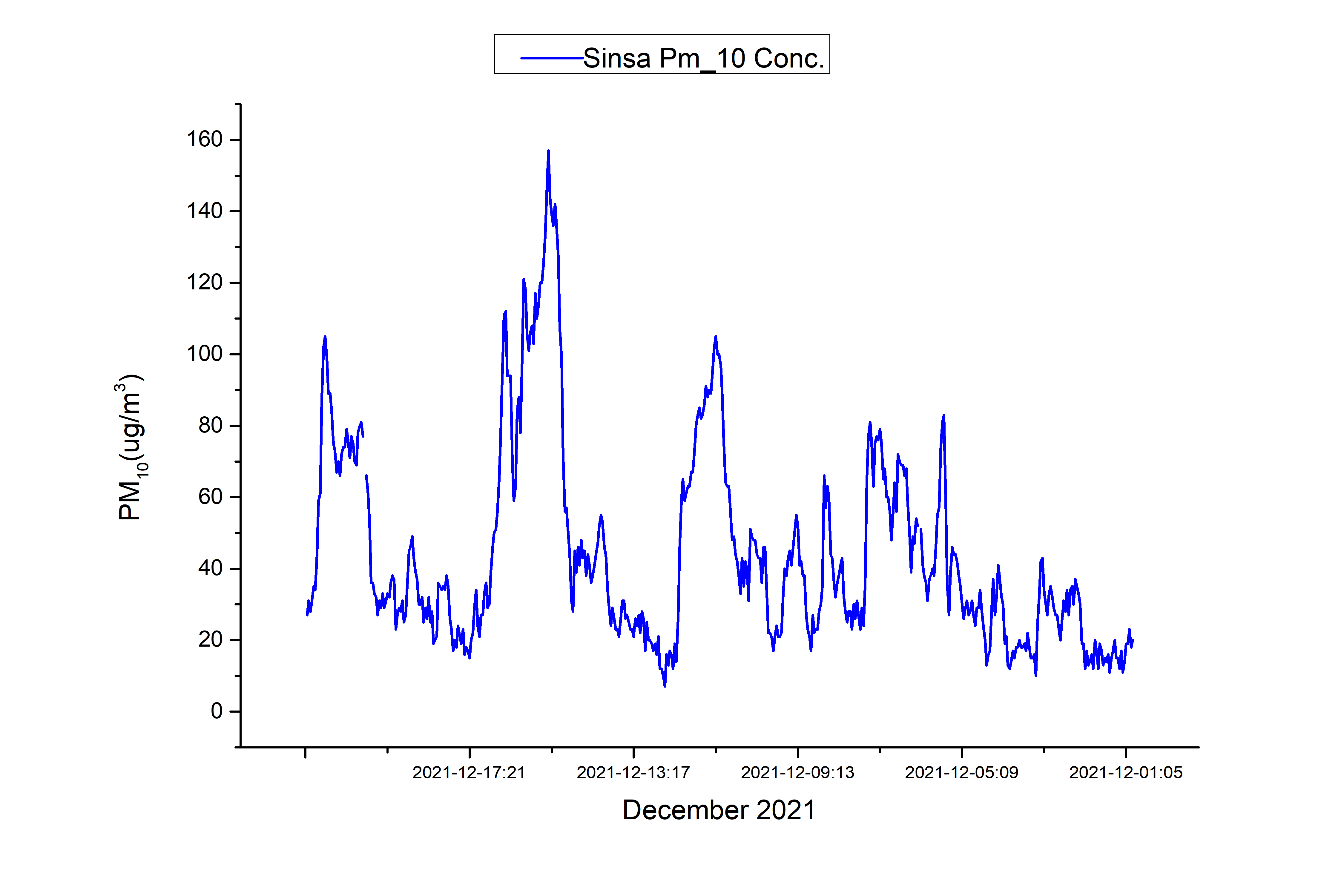


**Fig S3**- Time series of PM10 mass concentration (µg/m3) at each Subway station.


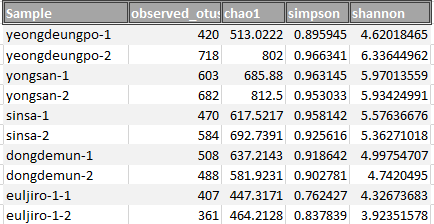


**Fig S4**- Alpha diversity measures for bacterial samples from each station collected from filter samples

**Fig S5**- The relative abundance of bacterial OTUs at the class level for each PM10 sample taken from Seoul subway stations.

**Fig S6**- The relative abundance of bacterial OTUs at the species level for each PM10 sample taken from Seoul subway stations.

**The Accession Number details of the NCBI submission**
